# Supplementary material for: Multi-ancestry genome-wide association study of major depression aids locus discovery, fine mapping, gene prioritization and causal inference
Source: Nat Genet. 2024 Jan 4;56(2):222–33. doi: 10.1038/s41588-023-01596-4 (PMC10864182; doi:10.1038/s41588-023-01596-4)
Supplement: Supplementary file 1 — Supplementary note and Figs. 1–21. [file 41588_2023_1596_MOESM1_ESM.pdf]

nature genetics

Article

https://doi.org/10.1038/s41588-023-01596-4

Multi-ancestry genome-wide association study of major depression aids locus discovery, fine mapping, gene prioritization and causal inference

In the format provided by the authors and unedited

Supplementary Information

Supplementary Note

Study Descriptions

A description of the following studies is available in our previous manuscript[1]: China Kadoorie Biobank (CKB), China, Oxford and Virginia Commonwealth University Experimental Research on Genetic Epidemiology cohort (CONVERGE), 23andMe cohort, Taiwan Major Depressive Disorder Study, Women's Health Initiative (WHI), Intern Health Study (IHS), UK Biobank (UKB), Army Study to Assess Risk and Resilience in Service members (Army-STARRS), and BioMe.

Additional notes for the Women's Health Initiative, Intern Health Study, UK Biobank and Army-STARRS studies:

Women's Health Initiative

The study was approved by the ethics committees at the Women's Health Initiative Coordinating Center, Fred Hutchinson Cancer Research Center, and at all 40 clinical centres[2].

Intern Health Study

This study was approved by the University of Michigan Medical School Institutional Review Board (HUM00033029)[3].

UK Biobank

UKB has approval from the North West Multi-centre Research Ethics Committee (MREC) as a Research Tissue Bank (RTB) approval. The approval was granted initially in 2011 and it is renewal on a 5-yearly cycle: hence UK Biobank successfully applied to renew it in 2016 and 2021 (https://www.ukbiobank.ac.uk/learn-more-about-uk-biobank/about-us/ethics).

Army-STARRS

Recruitment, consent, and data protection procedures were approved by the Human Subjects Committees of the Uniformed Services University of the Health Sciences for the Henry M. Jackson Foundation (the primary grantee), the Institute for Social Research at the University of Michigan (the organization implementing all Army STARRS surveys), and all other collaborating organizations. As in NSS, AAS respondents were additionally asked for consent to link Army and DoD administrative records to their SAQs and to participate in to-be-determined longitudinal follow-up studies. These procedures were approved by the Human Subjects Committee of all collaborating organizations[4].

Additional notes for the 23andMe cohort: Participants provided informed consent and participated in the research online, under a protocol approved by the external AAHRPP-accredited IRB, Ethical & Independent Review Services (E&I Review). Participants were included in the analysis on the basis of consent status as checked at the time data analyses were initiated.

Genes & Health

Genes & Health (GH) is a community based, long-term study of health and disease in British Bangladeshi and British Pakistani people in east London[5]. GH uses a population-based study design, as well as incorporated genomics analysis, electronic health record (EHR) data, and targeted recall-by-genotype (RbG) studies[5]. As of February 2020 GH had 38 899 volunteers, and by 2023 it aims to reach 100,000 volunteers participating in the study.

Bangladeshi and British Pakistani individuals (16 and over) living in or working in east London are invited to take part. Participants are recruited at mosques, libraries, GP surgeries and outpatient clinics by bilingual health researchers. Stage 1 participants all give consent to lifelong EHR linkage and donate saliva samples for genetic tests[5]. DNA was extracted from the Oragene (DNA Genotek) saliva system and stored from all Stage 1 volunteers. By late

2019, 50 000 samples from stage 1 volunteers were genotyped on the Illumina Infinium Global Screening Array v3.0 (with an additional 46 662 Multi-Disease variants)[5].

Data in volunteer questionnaires and EHR data was checked for data concordance and data with >99% concordance for gender and year of birth was retained. Data that could not be resolved with manual checking or data with clear data entry errors were removed[5].

GH is aiming to genotype and high-depth exome sequence up to 100 000 volunteer samples by 2023[5].

Cases and controls were defined on the participants' electronic medical records. Participants were defined as cases for MD if they had an ICD code for major depression and never had any of the following diagnoses: autism, bipolar disorder, dementia, Korsakoff psychosis, a manic episode, personality disorder, psychotic disorder, psychogenic fatigue, seasonal affective disorder or postpartum depression. Controls were those participants without an ICD code for major depressive disorder or any of the exclusion diagnoses and did not have a record of antidepressant prescription.

Biobank Japan

BioBank Japan (BBJ) is a prospective biobank that collected DNA and serum samples from 12 medical institutions in Japan between 2003 and 2008 and recruited approximately 200,000 participants (https://biobankjp.org/en/index.html)[6].

Participants were recruited on the basis of having a disease diagnosis of at least 1 out of 47 target diseases[7]. All study participants provided written informed consent. The mean age of participants at recruitment was 63.0 years of age, and 46.3% of the participants were female. Participants were mainly of Japanese ancestry[6]. Data on medical history, drug prescription reports and biomarkers have also been collected [6].

Initial Quality control of participants and genotypes in the initial BBJ

Genotyping of participants was performed using Illumina HumanOmniExpressExome BeadChip or a combination of the Illumina HumanOmniExpress and HumanExome BeadChip[8]. Variants with the following criteria were excluded: call rate <99%, *P* value for Hardy–Weinberg equilibrium $<1.0 \times 10^{-6}$, and number of heterozygotes <5[8]. In the GWAS, Eagle[9] was used for haplotype phasing without an external reference panel and Minimac3[10] was used for imputation. Variants with an Rsq ≥0.3 were used in the association analysis[8].

Further quality control in the 220 deep-phenotype GWAS data, used directly in the current study and conducted in the BBJ

The genotype data were further imputed with 1000 Genomes Project Phase 3 version 5 genotype data ($n = 2,504$) and Japanese whole-genome sequencing data ($n = 1,037$) using Minimac3 software[6]. Variants with an imputation quality of Rsq < 0.7 were excluded, resulting in 13,530,797 variants analysed in total[6].

A total of 3,893 MD cases were included in GWAS analysis, who had past medical history of depression based on ICD codes or had been prescribed antidepressants (ATC code: N06A) in BBJ. Meanwhile, 174,747 participants were included as controls. GWAS for binary traits (disease endpoints and medication usage) were performed using a generalised linear mixed model implemented in SAIGE (v.0.37), adjusting for age, age^2, sex, age × sex, $age^2 \times$ sex and the top 20 PCs[6].

Million Veteran Program

The Million Veteran Program (MVP) is one of the world's largest programs on genetics and health, containing genetic data of over 870,000 veteran partners since launching the program in 2011. MVP is also one of the most diverse cohorts in the world, collecting data from participants across the United States of America (https://www.research.va.gov/MVP/research.cfm). The MVP is an observational cohort

study combined with an electronic health record system, as well as a US Department of Veterans Affairs based mega-biobank.

Data are being collected from participants in the format of:

(1) questionnaires (a baseline survey on demographics, health status, medical history and a lifestyle survey on sleep, exercise, diet, and sense of wellbeing),

(2) through accessing electronic health records, and

(3) through a provided blood sample for genomic testing[11].

The study population is defined as active users of the Veteran Health Administration (VHA, large health care system) who are able to provide informed consent. 92.0% of the participants are male, between 50 and 69 years (55.0%). Self-reported race of 77.2% of the participants is white, and 13.5% is African-American[11]. Peripheral blood samples are collected by phlebotomists and processed plasma, buffy coat and DNA are stored in nitrogen freezers at The Department of Veterans Affairs Central Biorepository in Boston [11]. Genotyping (of approximately 459,777 samples) was performed using Affymetrix Axiom Biobank Array, with approximately 723K markers[12]. Quality control had been described in detail in Hunter-Zinck et al[12]. In brief, imputation was executed using IMPUTE2 and the 1000 Genomes Phase 3 reference panel. Autosomal markers with genotype missingness greater than 1% and all non-autosomal markers were removed. Samples with missingness of over 5% and closely related individuals were removed. For individuals of East Asian ancestry, the genotype data for biallelic SNPs were imputed using Minimac4 and a matched reference panel by the Sanger Institute. Indels and complex variants were imputed independently using the 1000 Genomes (1KG) phase 3 panel and merged in an approach similar to that employed by the UK Biobank.

For the current study, we have used summary statistics from individuals of African and East Asian ancestry from MVP. Classification as a case required at least one inpatient code or two or more outpatient codes for Major Depressive Disorder (MDD). Classification as a control required no record of inpatient or outpatient codes for MDD. Subjects with only one outpatient code for MDD were excluded from all analyses[13].

Australian Genetics of Depression Study (AGDS)

The Australian Genetics of Depression Study (AGDS) is one of the largest cohorts for studying genetic and psychosocial risk factors for depression[14]. For this study, more than 21,000 participants were recruited through two approaches: (1) through a nationwide recruitment based on pharmaceutical prescription history in the last 4.5 years and (2) through the Australian Department of Human Services and a media campaign (75% female, overall average age 43 years±15 years). Participants were asked to complete a self-reported online questionnaire assessing psychiatric history, clinical depression (using the Composite Interview Diagnostic Interview Short Form), responses to commonly prescribed antidepressants as well as voluntary questions assessing a range of traits relevant to psychopathology.

Participant enrollment was through the study website (https://www.geneticsofdepression.org.au/) hosted on a secure server (QIMR Berghofer). The website provided an information sheet about the study as well as a consent form for participants. Participants wishing to take part in the study were requested to sign the study informed consent and were asked to provide their name, age, and contact details. Participants' data were stored securely on the QIMR server[14]. To complete the questionnaire, participants were provided with a unique link to the questionnaires hosted on the Qualtrics website.

In addition to informed study consent, participants were asked to consent to provide access to their list of Medicare and Pharmaceutical Benefits Scheme (PBS) records for the previous 4.5 years. Consent was given by approximately 75% of the participants. Participants who did not consent to provide access to these records were still eligible to enroll to the study (around 25%).

Additionally, DNA samples were also collected from 75% of participants who agreed to provide their saliva samples via postal saliva kit. Saliva was self-collected by each consenting

participant using an Isohelix GeneFix GFX-02 2 mL saliva collector, and a sample tube as well as a signed consent form specific to the treatment of genetic information were returned by post. DNA was extracted from the saliva sample and stored in freezers[14].

An additional clinical trial cohort of 214 individuals (66% female; mean age = 51.3, SD = 12.5, range = 21–79; mean BMI = 27.7, SD = 5.6) from a mental health cohort from Deakin University's IMPACT Institute was also included in the study, in order to increase the sample size. All blood samples in this cohort were from individuals who provided signed informed consent for further unspecified use of their samples[14].

Genotyping of participants from the study cohorts was done using the Illumina Global Screening Array V.2.0. for 15,792 participants as of November 2021[14]. The AGDS GWAS only consisted of participants who met the DSM-5 criteria for MDD at some point in their lifetime , did not report a diagnosis of schizophrenia, bipolar disorder or attention deficit hyperactivity disorder, and had not participated in previous studies contributing to Psychiatric Genomics Consortium (PGC)[14] The final sample size for the GWAS was 13,104 cases from AGDS and 214 cases from the Deakin University samples. The controls were selected from another volunteer community sample from Queensland, Australia (QSkin cohort)[15]. The QSkin participants who reported never having been diagnosed with any psychiatric disorders were included as control subjects (n = 12,684). The GWAS was conducted using the SAIGE v. 0.39 [16]. Results were filtered on a minor allele frequency > 0.01 and R^2 INFO score > 0.6 [14].

Genetic Epidemiology Research on Adult Health and Aging (GERA)

Individual data of the GERA study was accessed via dbGaP under project ID of 18933. All study procedures were approved by the Institutional Review Board of the Kaiser Foundation Research Institute (https://bio-protocol.org/exchange/minidetail?type=30&id=9059082). The GERA study is a sub-study of the Kaiser Permanente (KP) Research Program on Genes,

Environment, and Health (RPGEH), which consisted of 103,006 adult members of Kaiser Permanente North California (KPNC), ranging from 18 to 100 years at enrollment. The study design for RPGEH and GERA has been described in detail elsewhere[17]. Briefly, adult members of KPNC were asked to complete a mailed survey, the respondents of which completed a broad written consent and provided a saliva sample for DNA extraction. GERA participants were also asked to self-report their race, ethnicity, nationality and religion in order to maximize the diversity of the cohort. As a result, the final cohort was formed by 19% non-European individuals and 81% randomly drawn non-Hispanic white individuals. Phenotypes on demographic and behavioural factors (e.g., gender, marital status, education, smoking, alcohol consumption) were derived from the RPGEH surveys. Data on the occurrence of health conditions in participants in the GERA Cohort had been derived from summarising ICD-9 coded diagnoses in Kaiser Permanente's electronic medical records. An algorithm that aggregates specific ICD-9 codes into appropriate diagnostic groups for selected conditions was applied to outpatient and inpatient databases (https://www.ncbi.nlm.nih.gov/projects/gap/cgi-bin/study.cgi?study_id=phs000674.v2.p2). The criterion for counting a condition as “present” for a participant is the occurrence of two or more diagnoses within a diagnostic category occurring on separate days.

DNA was extracted from participants’ saliva samples. The DNA extraction, genotyping, imputation, and relevant quality control procedures for the GERA cohort had been described elsewhere[18,19]. In short, GERA individuals were genotyped separately on four ethnic-specific arrays (Affymetrix Axiom) according to their self-reported ancestry. There were 84,430 participants assayed on the non-Hispanic European array, 8,043 on the East Asian array, 5,779 on the African array and 11,585 on the Hispanic/Latin American array, respectively. The quality control for samples included DishQC score (DQC) of no less than 0.82 and sample call rate of no less than 97%. DishQC is a measure of the contrast between the AT and GC signals assayed in non polymorphic test sequences (Affymetrix White Paper). It provides a type of signal-to-noise figure of merit that is well correlated with sample call rate, allowing for prediction of successful samples. Afterwards, genotypes were imputed with the 1000 Genomes (October 2014 release) as a reference panel, implemented per array by IMPUTE2 v2.3.1[20,21].

ICD-9 billing codes were acquired for the study participants, which were summarized to define health conditions. ICD-9 codes of 296.2 (including 296.20 – 296.26), 296.3 (including 296.30 – 296.36) and 296.82 were considered as depression. Participants with two or more occurrences of depression diagnostic codes on separate days were defined as cases for depression.

The genetic ancestry based on the genotype imputation data was calculated by Graf-pop [22]. Due to admixture between pre-defined major ancestry groups, 1,200 participants were removed. In total, there were 226 MD cases and 3,722 controls of East Asian ancestry, 206 cases and 1,598 controls of African ancestry, 415 cases and 2,808 controls of Hispanic/Latin American ancestry. GWAS were implemented for these three ancestry groups separately by PLINK2 on the imputed dosage data, adjusting for sex, age and the first 10 PCs.

Jackson Heart Study (JHS)

Data of the Jackson Heart Study (JHS) was accessed via dbGaP under project ID of 18933. The study was approved by the Institutional Review Board of the National Institutes of Health and the study protocol was approved by the Institutional Review Boards of the participating JHS institutions, including the University of Mississippi Medical Center, Jackson State University and Tougaloo College[23]. The participants with available depression phenotypes from a sub-study of JHS with genotyped dataset were analysed for the current study (dbGaP study accession: phs001356.v1.p2). Study designs for the JHS study have been described elsewhere[24]. In short, the JHS is a large, community-based, observational study, which recruited 5,301 participants from among the non-institutionalized African-American adults from urban and rural areas of the three counties (Hinds, Madison, and Rankin) that make up the Jackson, MS, metropolitan statistical area (MSA). Participants provided extensive medical and social history and had an array of physical and biochemical measurements and diagnostic procedures during a baseline examination (2000-2004) and two follow-up examinations (2005-2008 and 2009-2012). For current depression (past week symptoms), cases were identified by a 20-item CES-D score of 16 or greater.

Study participants were genotyped by the Illumina Human Exome BeadChip v1.1 array and then imputed to the 1000 Genomes Phase 3 African reference panel. Samples with genotyping call rate of less than 95% and variants which were successfully genotyped in less than 95% of samples were excluded. Relatedness coefficients were calculated by KING[25]. Related individuals up to 2nd degree relatedness were randomly excluded (kinship > 0.0884).

A total of 299 depression cases and 990 controls were included in our GWA for the JHS study. Logistic regressions adjusting for age, gender, recruitment type and first 20 PCs were implemented in PLINK2. Following the analyses, variants with imputation R squared of less than 0.7 or a minor allele count of less than 50 were excluded.

The Drakenstein Child Health Study (DCHS)

The DCHS is a population-based birth cohort study in the Drakenstein area in Paarl, a peri-urban area 60 km outside Cape Town, South Africa. Data collection occurred at two clinics (maternal data) as well as at a central hospital (newborn outcomes) in the Drakenstein area between March 2012 to March 2015. The study was approved by the faculty of Health Sciences, Human Research Ethics Committee, University of Cape Town (401/2009), Stellenbosch University (N12/02/0002) and the Western Cape Provincial Health Research committee (2011RP45)[26]. Participants were enrolled in the DCHS at 20 to 28 weeks' gestation upon presenting for antenatal booking and followed longitudinally throughout pregnancy until at least five years postnatally. Maternal, paternal and child health are investigated through longitudinal measurements of risk factors in seven areas (environmental, infectious, nutritional, genetic, psychosocial, maternal and immunological) that may impact on child health[27,28]. More detailed information on the DCHS can be found on the study website (http://www.paediatrics.uct.ac.za/scah/dclhs). Exclusion criteria for the DCHS were minimal in order to maximise generalisability, and focused primarily on those individuals who did not live in the region (and thus could not be readily followed up) or those who were intending to move out of the district within the first year. Exclusion criteria

for cases and controls consisted of women who had stillbirths, infant deaths, gave birth to twins/triplets, were diagnosed with lifetime bipolar disorder or psychosis[29].

DNA was extracted from whole blood using the QIAsymphony DSP DNA Midi kit and protocol (Qiagen, Hilden, Germany). Genome-wide SNP genotyping was conducted using either the Infinium PsychArray or Global Screening Array-24 BeadChip (Illumina). Standard quality control of the genome-wide data was performed using PLINK removing individuals with >5% missing data and removing one in each pair of related individuals with an IBD proportion >0.12 (indicating cousins or a closer relation). The DCHS researchers removed SNPs with call rates <95%, MAF <0.05 and deviation from Hardy–Weinberg proportions ($P < 1 \times 10^{-6}$ in controls and $P < 1 \times 10^{-10}$ in cases). To evaluate population stratification, PC eigenvectors of the genetic relationship matrix were calculated by using about 50 000 independent SNPs. SNPs in LD (r^2=0.075) were excluded to calculate PCs. Dimensional plots of the PCs were also used to remove outliers. The Faculty of Health Sciences human research ethics committee of the University of Cape Town (UCT) approved this study.

We acquired individual-level genotype imputed data from the DCHS researchers. Depression cases were defined by BDI-II score (no less than 20 points). Controls were defined with both BDI-II score and EPDS score (BDI-II score of less than 20 points and EPDS score of less than 13 points). There were 139 cases and 346 controls in our analysis. Logistic regressions were run by PLINK2 adjusting for the first 20 PCs, recruitment site, age of enrolment for controls or age of assessment for cases.

Vanderbilt University Biobank

The Vanderbilt University Medical Center’s (VUMC, Nashville, USA) Biobank (BioVU) includes more than 285,000 patients seen at the VUMC, whose DNA was extracted (from whole blood) and linked to de-identified electronic health records (EHR) data spanning 1990–2017[30]. A detailed consent form, including information on policies, data sharing and privacy is provided to patients seen at the clinic at VUMC. Signed informed consent of the

patient is required to deposit new blood samples left over from clinical care to the BioVU Biobank. BioVU is overseen and approved by the VUMC Institutional Review Board.

Genotyping was performed on a subset of BioVU patients ($n = 24,262$) using the Illumina MEGA^{EX} platform, which contains more than two million markers. Quality control was done as described in Ruderfer et al. 2020[31]. Briefly, samples with greater than 2% missingness or abnormal heterozygosity were removed. Variants with greater than 2% missingness or Hardy-Weinberg equilibrium p-value $< 5 \times 10^{-5}$ were excluded. SNPs with minor allele frequency less than 2% and SNPs not genotyped in HapMap2 were also excluded. Imputation was performed using the pre-phasing/imputation stepwise approach in IMPUTE4 / SHAPEIT, using 1000 genomes phase I reference panel. Variants with INFO <0.3 were excluded [31].

A subset of SNPs in linkage disequilibrium was used to calculate relatedness and principal components of ancestry using multidimensional scaling in PLINK v1.9[32]. One individual from pairs of highly related individuals (pi_hat > 0.1) were randomly excluded. Samples were genotyped in five batches, and variants were removed if allele frequencies differed significantly ($P < 5 \times 10^{-5}$) between any batch and the rest of the sample[32]. Finally, multiallelic and structural variants were filtered, dosage data was converted to hard genotype calls, and variants with certainty <0.9 or INFO < 0.95 were excluded, resulting in 5,218,407 high quality SNPs across the autosomes[31,32].

The International Classification of Diseases, 9th edition (ICD-9) billing codes of participants in the EHR were mapped to phecodes according to the Phecode Map v1.2 as implemented in the PheWAS R package v0.12 [15], which are the higher order representations of disease categories. Participants were assigned as cases for major depression if they had at least two different ICD-9 codes that mapped to phecode ‘296.2’, or if they had at least two separate occurrences (i.e., on different days) of a single ICD-9 code that mapped to the phenode ‘296.2’. The control group excluded participants with only one component ICD-9 codes, or with one or more ICD-9 codes that mapped to related phecodes (phecodes 295 - 306.99, as defined by the Phecode Map v1.2)38. In GWAS, linear mixed models were fitted by SAIGE adjusting for the first 10 PCs, age and sex.

Hispanic Community Health Study/Study of Latinos (HCHS/SOL)

HCHS/SOL is a prospective, multicenter, population-based cohort study of Hispanic/Latin American adults in the United States. It recruited 16,000 Hispanic/Latin American participants from Bronx, Chicago, Miami and San Diego under a two-stage area probability sampling[33]. All participants went through thorough baseline examination, which lasts for 7 hours on average. Signed informed consent was obtained when participants arrived at assessment centres, followed by fasting state measurements (e.g. anthropometry, phlebotomy, 2-hour glucose load) and several other measurements (e.g., EGC, seated blood pressure). Participants were also administered with a questionnaire collecting their socio-demographic status, medical history, substance use, wellbeing, etc.[34]. The HCHS/SOL study was approved by institutional review boards at participating centres, and written informed consent was obtained from all participants.

DNA extracted from blood was genotyped on an Illumina custom array, SOL HCHS Custom 15041502 B3, consisting of the Illumina Omni 2.5M array (HumanOmni2.5-8v1-1) and ~150,000 custom SNPs selected to include ancestry-informative markers, variants characteristic of Amerindian populations, previously identified GWAS hits, and other candidate-gene polymorphisms. Genotype imputation was performed with the 1000 Genomes Project phase 1 reference panel implemented by SNAPEIT2 and IMPUTE2[35].

Depressive symptoms were assessed during the baseline assessment with the Andresen version of the 10-item Center for Epidemiology Studies of Depression Scale (CES-D-10), which reflected core symptoms of depression in the past week[36]. The CES-D-10 scores were curated into a binary phenotype, where participants with scores of no less than 10 were defined as cases (N = 3,979) and those with scores of 6 or below were defined as controls (N = 6,499). Mixed-effect model logistic regressions adjusting for log of sampling weight, recruiting centre age, sex, highest education attained, genetic subgroup and the first 5 PCs were conducted by GENESIS[36,37].

Detroit Neighborhood Health Study (DNHS)

The DNHS is an ongoing, longitudinal epidemiologic study investigating correlates of PTSD and other mental disorders in the city of Detroit. It recruited adults (18 years or above) from the Detroit population. The Institutional Review Board of the University of Michigan reviewed and approved the study protocol (https://www.ncbi.nlm.nih.gov/projects/gap/cgi-bin/study.cgi?study_id=phs000560.v1.p1). Initially, 1,547 households were randomly drawn from the city of Detroit under a probability sample. Then an interview was conducted with one random individual from each household. Participants underwent a 40-minute assessment consisting of questions on socio-demographic characteristics, major depressive disorder (MDD), and generalized anxiety disorder (GAD)[38]. MD was scored using the Patient Health Questionnaire (PHQ-9)[39] and DSM-IV criteria[40]. The Institutional Review Board at the University of Michigan and the University of North Carolina Chapel Hill approved this study.

DNA for GWAS analysis was isolated from peripheral blood or saliva. Study participants were genotyped with Illumina HumanOmniExpress array and imputation was conducted based on the 1000 Genomes phase 3 data. Relatedness was estimated using the IBS function in PLINK 1.9. From each pair with relatedness $\pi\hat{} > 0.2$, one individual was removed from further analysis, retaining cases where possible. Principal components were calculated based on the smartPCA algorithm in EIGENSTRAT[41].

A total of 58 cases, which were defined at the baseline visit, and 436 controls of African ancestry were included in our MD GWAS for the DNHS cohort. Individual level genotype imputed data and phenotype data were shared with us. Logistic regressions were implemented by PLINK2 with imputed dosage data, adjusting for sex, age at baseline assessment, and the first 20 PCs.

Prevention Intervention Research Center (PIRC) 1st Generation Trial

This study was a trial designed and conducted by the Prevention Intervention Research Center at Johns Hopkins University. A total of 2,311 youth entering first grade in 1985 or 1986 in 19 primary public schools, which were selected from five areas to represent the socio-demographic diversity in the northeastern quadrant of Baltimore in 1985, were recruited[42]. The trial was initially aimed at assessing the immediate effects of two universal, first-grade preventive interventions (i.e., classroom-centered intervention vs family-school partnership intervention) on the proximal targets of poor achievements, concentration problems, aggression and shy behaviours, which were known as early risk behaviours for later substance use/abuse, affective disorder and conduct disorder[43]. Twenty five years later, 65% of the surviving cohort (n=1,434) participated in a follow-up interview that inquired about their general and mental health, including alcohol, tobacco, and other drug involvement. Lifetime history of major depressive episode was measured using the Composite International Diagnostic Interview-University Version, CIDI-UM (World Health Organization 1997). The study protocol was approved by the institutional review board for protection of human subjects at Johns Hopkins University [42].

Participants were asked about their willingness to donate a blood sample. If unwilling and/or unable to donate blood, they were then asked if they would donate a saliva sample instead. DNA was extracted from blood or saliva samples, then quantitated and genotyped using Affymetrix 6.0 microarray (Santa Clara, CA, USA)[44]. Genotypes were imputed to the TopMed using the Michigan Imputation Server[45]. The resulting variants imputed with an INFO score of less than 0.8 were removed. Genome-wide logistic regressions were conducted by R (version 3.6.1) for 52 cases and 547 controls of African ancestry, adjusting for participant age, sex, intervention status (control vs exposure to intervention) and the first 20 PCs.

Mexican Adolescent Mental Health Survey (MAMHS)

MAMHS is a multistage probability survey of 3,005 adolescents aged 12-17 years residing in Mexico City. Interviews were conducted in the homes of the invited participants in 2005. Signed informed consents were obtained from parents or legal guardians, and assent of the

participating adolescents were obtained as well[46]. Lifetime depression was defined by the MDE DSM IV criteria, as with the computer-assisted version of adolescent CIDI.

DNA was extracted from exfoliated oral cavity cells and afterwards genotyped with the Illumina Global Screening Array (GSA). Imputation was carried out using the Michigan Imputation server using Minimac4 and the 1000 g-phase-3-v5 (hg19) full reference panels [47]. Variants with imputation INFO of less than 0.7, minor allele frequency of no larger than 0.01, missingness no less than 0.05 or with Hardy-Weinburgh P value of no larger than 1×10^{-6} were excluded. Participants with genotype missing rate of no less than 0.05 or heterozygosity outliers (> 3 standard deviation away from the mean) were excluded, leaving 105 cases and 996 controls of admixed Hispanic/Latin American ancestry for GWA analysis. Mixed-effect model logistic regressions were implemented by SAIGE [16] adjusting for age, sex and the first 20 PCs.

Pregnancy Outcomes, Maternal and Infant Study (PrOMIS)

The PrOMIS cohort is a prospective cohort aimed at understanding the life course and intergenerational effects of interpersonal violence and other forms of trauma among Peruvian women. Between 2012 and 2015, participants were recruited from prenatal care clinics at the Instituto Nacional Materno Perinatal (INMP) in Lima, Peru. A structured questionnaire including maternal socio-demographic, lifestyle characteristics, medical and reproductive histories, and mental health symptoms was completed by an interview with trained research staff. Depression was assessed with the Patient Health Questionnaire (PHQ-9), which enquired about depressive symptoms for the 2-week period prior to the interview. The institutional review boards of the INMP and the Office of the Human Research Administration, Havard T.H. Chan School of Public Health approved all procedures used in the study[48,49].

Genotyping was conducted on the Illumina Multi-Ethnic Global Chip. Imputation was conducted with the 1000 Genomes phase 3 data[50]. GWAS quality control and imputation was performed to the published PGC procedures[41]. PC-related and PC-Air was employed in

identifying and excluding related individuals, and calculating principal components[51]. There were 1,076 MD cases and 2,328 controls for the MD GWAS. GWA logistic regressions were conducted by PLINK, adjusting for the first 10 PCs.

Multi-ancestry linkage-disequilibrium reference panel

In order to facilitate multi-ancestry analyses, a multiple-ancestry LD reference panel was constructed by randomly drawing 10k participants from the UK Biobank. The proportions of individuals from each of the four major ancestries (European, African, East Asian and South Asian) in the multiple ancestry LD reference panel was matched to the proportions of ancestries in our multi-ancestry MD GWAS on the effective sample size scale. Hispanic/Latin American descent participants were omitted from the multiple ancestry LD reference due to their complex structure and low representation in the UK Biobank data. As a result, there were 7,658 participants of European (76.58%), 1,285 of African (12.85%), 891 of East Asian (8.91%) and 166 participants of South Asian (1.66%) ancestry in the multi-ancestry LD reference panel.

Sensitivity Analyses

In order to investigate the potential impact on results brought about by the heterogeneous outcome definitions used across studies, we conducted additional ancestry-specific meta-analyses and multi-ancestry meta-analyses restricted to studies which fulfilled the clinical definition of depression.

Furthermore, multiple studies were population-based and consequently presented with a small proportion of cases relative to the number of controls. This can be adjusted for analytically, for example by using SAIGE[53]. Among all studies, 18 had a case-control ratio of less than 0.25 and did not account for this analytically. Therefore, we conducted another sensitivity multi-ancestry meta-analysis excluding these studies. We also excluded one study with participants of adolescent age (MAMHS).

To compare the results from the sensitivity and main analysis on the effect estimates of genome-wide significant SNVs, we computed Pearson's correlation coefficient.

We also applied a multi-ancestry meta-analysis method, Meta-Regression of Multi-Ethnic Genetic Association (MR-MEGA)[53,62]. The MR-MEGA pipeline assumes that common causal variants are shared across ancestries, and the ancestry heterogeneity is caused by differences in allele frequencies of the causal variants. Thus, it models the allelic effect heterogeneity based on allele frequencies in individual GWAS summary statistics and conducts meta-analysis adjusting for the ancestry heterogeneity. For this, we first derived a matrix of mean pairwise allele frequency differences between GWASs. Afterwards we implemented multi-dimensional scaling of the distance matrix to derive axes of genetic variation, which represent ancestral PCs. Subsequently, the allelic effect estimates across GWASs were modelled in a linear regression framework, weighted by the inverse variance of the effect estimates, with the first three axes of genetic variation as covariates. SNVs in each GWAS summary statistics were filtered based on the aforementioned criteria. We corrected the meta-regression association P values for inflation due to residual structure between GWAS using genomic control adjustment (λ_{GC} = 1.287). SNVs with P values of smaller than 5×10^{-8} were considered as significant. Afterwards, GWAS significant SNVs were processed by the aftermentioned steps for defining independent loci by flanking genomic interval mapping 250kb upstream and downstream of each lead SNV.

STROBE-MR

STROBE-MR checklist of recommended items to address in reports of Mendelian randomization studies[52,53]

Item No.	Section	Checklist item	Page No.	Relevant text from manuscript
1	TITLE and ABSTRACT	Indicate Mendelian randomization (MR) as the study's design in the title and/or the abstract if that is a main purpose of the study	8	Mendelian Randomisation showed a bidirectional relationship with BMI exclusively in samples of European ancestry.
	INTRODUCTION			
2	Background	Explain the scientific background and rationale for the reported study. What is the exposure? Is a potential causal relationship between exposure and outcome plausible? Justify why MR is a helpful method to address the study question	9	This heterogeneity could impact on findings of genetic studies when evaluating causal effects of risk factors for MD. Previous studies in samples of European ancestry reported genetic correlations and causal relationships between MD and cardiometabolic outcomes.
3	Objectives	State specific objectives clearly, including pre-specified causal hypotheses (if any). State that MR is a method that, under specific assumptions, intends to estimate causal effects	9f	Thus, investigating causal relationships using Mendelian Randomization in diverse ancestry groups and in different disease subtypes is important to ensure generalisability and to distinguish between biological and societal mechanisms underlying the relationship between a risk factor and the disease.

				Finally, we explored bi-directional causal links between MD and cardiometabolic traits.
	METHODS			
4	Study design and data sources	Present key elements of the study design early in the article. Consider including a table listing sources of data for all phases of the study. For each data source contributing to the analysis, describe the following:		
	a)	Setting: Describe the study design and the underlying population, if possible. Describe the setting, locations, and relevant dates, including periods of recruitment, exposure, follow-up, and data collection, when available.	19	We included data from 21 studies with ancestrally diverse participants where measurements were taken from distinct samples. Details including study design, genotyping and imputation methods and quality control for these studies had been described by previous publications (Supplementary Material). All participants have provided informed consent. All studies obtained ethical approvals from local ethics review boards.

b)	Participants: Give the eligibility criteria, and the sources and methods of selection of participants. Report the sample size, and whether any power or sample size calculations were carried out prior to the main analysis	19, 26	We also included previously published studies of MD using data from ancestrally European participants, including PGC-MDD (N cases = 246,363, N controls = 561,190)[54] and the AGDS (N cases = 12,123, N controls = 12,684) to conduct a multi-ancestry meta-analysis of MD). The total sample size of the multi-ancestry meta-analysis was 1,820,927 (N cases = 342,883; Neffhalf = 485,107). 70.1% of participants (effective sample size) were of European ancestry and 8.2% East Asian, 11.8% African and 1.5% South Asian ancestry and 7.9% Hispanic/Latin American.<br><br>For individuals of European ancestry, the UK Biobank was used to select instruments for BMI, fasting glucose (FG), high-density lipoprotein (HDL), low-density lipoprotein (LDL), systolic blood pressure (SBP), and triglycerides (TG). SBP summary data were obtained from the UK Biobank for individuals of African and South Asian ancestry and Hispanic/Latin American participants. For samples of African, East Asian and South Asian ancestry and the Hispanic/Latin American group, a meta-analysis was performed using METAL[55] with inverse variance weighting using the UK Biobank and the following consortia: GIANT[56] for BMI, MAGIC[57] for FG, GLGC[58] for HDL, LDL, and TG, and BBJ[6,58] for SBP in samples of East Asian ancestry.

		c)	Describe measurement, quality control and selection of genetic variants	26	We performed a bi-directional two-sample MR analysis using the TwoSampleMR R package[59] to test possible causal effects between MD and six cardiometabolic traits.<br><br>Genome-wide significance (P = 5x10-8) was used as the threshold to select instrumental variables for the exposures. However, if less than 10 variants were available, a suggestive threshold (P = 5x10-6) was used to select instrumental variables.
		d)	For each exposure, outcome, and other relevant variables, describe methods of assessment and diagnostic criteria for diseases	19	A range of measures were used to define depression, including structured clinical interviews, medical care records, symptom questionnaires and self-reported surveys.
		e)	Provide details of ethics committee approval and participant informed consent, if relevant	19	All participants have provided informed consent. All studies obtained ethical approvals from local ethics review boards.
5	Assumptions		Explicitly state the three core IV assumptions for the main analysis (relevance, independence and exclusion restriction) as well assumptions for any additional or sensitivity analysis	26	We followed the three main IV assumptions for the analysis: i, relevance: the IV is associated with the risk factor of interest; ii, independence: the IV is not associated with confounders; exclusion: the IV is only associated with the outcome through the exposure.<br><br>MR-Egger regression intercept and MR heterogeneity tests were conducted as additional sensitivity analyses. In case of significant heterogeneity, the MR-PRESSO (Pleiotropy RESidual Sum and

				Outlier) global test was used to remove genetic variants based on their contribution to heterogeneity).
6	Statistical methods: main analysis	Describe statistical methods and statistics used		
	a)	Describe how quantitative variables were handled in the analyses (i.e., scale, units, model)	26	The genetic associations with quantitative variables were estimated with respect to the scale, units, and models defined in the original studies.
	b)	Describe how genetic variants were handled in the analyses and, if applicable, how their weights were selected	26	Genome-wide significance (P = 5x10-8) was used as the threshold to select instrumental variables for the exposures. However, if less than 10 variants were available, a suggestive threshold (P = 5x10-6) was used to select instrumental variables.
	c)	Describe the MR estimator (e.g. two-stage least squares, Wald ratio) and related statistics. Detail the included covariates and, in case of two-sample MR, whether the same covariate set was used for adjustment in the two samples	26	We used five different MR methods: inverse variance weighted (IVW), MR-Egger, weighted median, simple mode, and weighted mode. The IVW estimates were reported as the main results due to their higher statistical power while the other tests were used to assess the consistency of the estimates across different methods.
	d)	Explain how missing data were addressed	26	We only included IVs which were present in both datasets (exposure and the outcome).

		e) If applicable, indicate how multiple testing was addressed		
7	Assessment of assumptions	Describe any methods or prior knowledge used to assess the assumptions or justify their validity	9	Previous studies in samples of European ancestry reported genetic correlations and causal relationships between MD and cardiometabolic outcomes.
8	Sensitivity analyses and additional analyses	Describe any sensitivity analyses or additional analyses performed (e.g. comparison of effect estimates from different approaches, independent replication, bias analytic techniques, validation of instruments, simulations)	26	The IVW estimates were reported as the main results due to their higher statistical power while the other tests were used to assess the consistency of the estimates across different methods. MR-Egger regression intercept and MR heterogeneity tests were conducted as additional sensitivity analyses. In case of significant heterogeneity, the MR-PRESSO (Pleiotropy RESidual Sum and Outlier) global test was used to remove genetic variants based on their contribution to heterogeneity).
9	Software and pre-registration			
		a) Name statistical software and package(s), including version and settings used	26	We performed a bi-directional two-sample MR analysis using the TwoSampleMR R package (v0.5.6).<br>We used the following criteria for clumping: r2=0.001 and a 10,000-kb window. The following information was used in both the exposure and outcome data: SNP ID, effect size, effect allele, other allele, effect allele frequency, and p value.

		b)	State whether the study protocol and details were pre-registered (as well as when and where)		Not Applicable
	RESULTS				
10	Descriptive data				
		a)	Report the numbers of individuals at each stage of included studies and reasons for exclusion. Consider use of a flow diagram	26	To avoid sample overlap, the datasets used to define instrumental variables for the cardiometabolic traits were excluded from the MD genome-wide association statistics used for the MR analyses conducted with respect to each ancestry group.
		b)	Report summary statistics for phenotypic exposure(s), outcome(s), and other relevant variables (e.g. means, SDs, proportions)	21	We combined data from 71 cohorts with diverse ancestry using an inverse-variance weighted fixed-effects meta-analysis in METAL[55]. λ and λ1000 were calculated, which were 1.687 and 1.001, respectively. The LDSC intercept was also calculated with the multi-ancestry LD reference panel, which was 1.019 (SE = 0.011). We adjusted the test statistics from the multi-ancestry meta-analysis using the LDSC intercept of 1.019. Only variants present in at least two studies were retained for further analysis, yielding a total of 22,941,580 variants.

	c)	If the data sources include meta-analyses of previous studies, provide the assessments of heterogeneity across these studies	21, 25	We also performed heterogeneity analysis with METAL to assess whether observed effect sizes (or test statistics) are homogeneous across samples.<br>Heterogeneity analyses were also performed.
	d)	For two-sample MR:<br>i. Provide justification of the similarity of the genetic variant-exposure associations between the exposure and outcome samples<br>ii. Provide information on the number of individuals who overlap between the exposure and outcome studies	26	MR-Egger regression intercept and MR heterogeneity tests were conducted as additional sensitivity analyses. In case of significant heterogeneity, the MR-PRESSO (Pleiotropy RESidual Sum and Outlier) global test was used to remove genetic variants based on their contribution to heterogeneity92).<br>To avoid sample overlap, the datasets used to define instrumental variables for the cardiometabolic traits were excluded from the MD genome-wide association statistics used for the MR analyses conducted with respect to each ancestry group.
11	Main results			

a)	Report the associations between genetic variant and exposure, and between genetic variant and outcome, preferably on an interpretable scale	14	Our results indicated a positive, bi-directional relationship between MD and BMI (MD->BMI: β = 0.092, 95% CI = 0.024-0.161, P = 8.12×10-3, BMI->MD: β = 0.138, 95% CI = 0.097-0.180, P = 6.88×10-11) (Figure 4, Supplementary Table 13). This bi-directional relationship was exclusively observed in samples of European ancestry (P > 0.1 in all other groups). MD was also causal for other indicators of unfavourable metabolic profiles in samples of European ancestry: triglycerides (TG, positive effect; β = 0.116, 95% CI = 0.070-0.162, P = 7.93×10-7), HDL (negative effect; β = -0.058, 95% CI = -0.111- -0.006, P = 0.029), and LDL cholesterol (positive effect; β = 0.054, 95% CI = 0.012-0.096, P = 0.011). The effects remained significant after removing the variants contributing to the possible heterogeneity bias observed through the MR-PRESSO global test (Supplementary Table 13B). In samples of East Asian ancestry, on the other hand, we found a negative causal association between TG and MD (β = -0.127, 95% CI = -0.223- -0.032, P = 9.22×10-3). Moreover, MD showed a positive causal association with systolic blood pressure (SBP) (β = 0.034, 95% CI = 0.009-0.059, P = 7.66×10-3). In samples of African ancestry, SBP had a positive causal association with MD (β = 0.080, 95% CI = 0.026-0.133, P = 3.43×10-3).

**b)**	**Report MR estimates of the relationship between exposure and outcome, and the measures of uncertainty from the MR analysis, on an interpretable scale, such as odds ratio or relative risk per SD difference**	**14**	**Our results indicated a positive, bi-directional relationship between MD and BMI (MD->BMI: β = 0.092, 95% CI = 0.024-0.161, P = 8.12×10-3, BMI->MD: β = 0.138, 95% CI = 0.097-0.180, P = 6.88×10-11) (Figure 4, Supplementary Table 13). This bi-directional relationship was exclusively observed in samples of European ancestry (P > 0.1 in all other groups). MD was also causal for other indicators of unfavourable metabolic profiles in samples of European ancestry: triglycerides (TG, positive effect; β = 0.116, 95% CI = 0.070-0.162, P = 7.93×10-7), HDL (negative effect; β = -0.058, 95% CI = -0.111- -0.006, P = 0.029), and LDL cholesterol (positive effect; β = 0.054, 95% CI = 0.012-0.096, P = 0.011). The effects remained significant after removing the variants contributing to the possible heterogeneity bias observed through the MR-PRESSO global test (Supplementary Table 13B). In samples of East Asian ancestry, on the other hand, we found a negative causal association between TG and MD (β = -0.127, 95% CI = -0.223- -0.032, P = 9.22×10-3). Moreover, MD showed a positive causal association with systolic blood pressure (SBP) (β = 0.034, 95% CI = 0.009-0.059, P = 7.66×10-3). In samples of African ancestry, SBP had a positive causal association with MD (β = 0.080, 95% CI = 0.026-0.133, P = 3.43×10-3).**
**c)**	**If relevant, consider translating estimates of relative risk into absolute risk for a meaningful time period**		

		d)	Consider plots to visualize results (e.g. forest plot, scatterplot of associations between genetic variants and outcome versus between genetic variants and exposure)	42	Figure 4. Bi-directional Mendelian Randomization analyses between major depression and cardiometabolic outcomes.
12	Assessment of assumptions				
		a)	Report the assessment of the validity of the assumptions	14	Additionally, no pleiotropy was observed (Supplementary Table 13).
		b)	Report any additional statistics (e.g., assessments of heterogeneity across genetic variants, such as I^2, Q statistic or E-value)	14	The effects remained significant after removing the variants contributing to the possible heterogeneity bias observed through the MR-PRESSO global test (Supplementary Table 13B).
13	Sensitivity analyses and additional analyses				
		a)	Report any sensitivity analyses to assess the robustness of the main results to violations of the assumptions	14	The effects remained significant after removing the variants contributing to the possible heterogeneity bias observed through the MR-PRESSO global test (Supplementary Table 13B). Additionally, no pleiotropy was observed (Supplementary Table 13).

	b) Report results from other sensitivity analyses or additional analyses		
	c) Report any assessment of direction of causal relationship (e.g., bidirectional MR)	14	We assessed bi-directional causal relationships between MD and cardiometabolic traits using ancestry-specific two-sample Mendelian Randomisation analyses.
	d) When relevant, report and compare with estimates from non-MR analyses		
	e) Consider additional plots to visualize results (e.g., leave-one-out analyses)		
**DISCUSSION**			

14	Key results	Summarize key results with reference to study objectives	17	Previous Mendelian Randomization (MR) studies conducted in populations of European ancestry suggested a causal relationship of higher BMI increasing the odds of depression. To our knowledge, evidence of a reverse causal association (i.e., MD increases the odds of higher BMI) had not been previously reported[60]. We also observed that the genetic liability to MD was associated with higher triglyceride levels, lower HDL cholesterol and higher LDL cholesterol in subjects of European ancestry, which were not significant in the only previous MR study of smaller statistical power. In other ancestry groups, no significant relationship between BMI and MD was observed. Moreover, our MR analyses showed an effect of reduced triglycerides on increasing odds of MD in participants of East Asian ancestry. Therefore, we provide further evidence for an opposite direction of effect for the relationship between MD and metabolic traits in European and East Asian ancestry groups.
15	Limitations	Discuss limitations of the study, taking into account the validity of the IV assumptions, other sources of potential bias, and imprecision. Discuss both direction and magnitude of any potential bias and any efforts to address them	17	Additionally, our bidirectional MR analysis tested the relationships between MD and cardiometabolic traits. When testing MD as the exposure, the results should be interpreted as the effect of MD genetic liability and not as the effect of MD itself. Finally, although we conducted several sensitivity analyses, we cannot exclude that the results of MR analyses may be affected by unaccounted violations of MR assumptions.

**16**	**Interpretation**				
		**a)**	**Meaning: Give a cautious overall interpretation of results in the context of their limitations and in comparison with other studies**	**17**	**Instead of generalising findings about depression risk factors across populations, further studies are needed to understand how genetic and environmental factors contribute to the complex relationships across diverse ancestry groups.**
		**b)**	**Mechanism: Discuss underlying biological mechanisms that could drive a potential causal relationship between the investigated exposure and the outcome, and whether the gene-environment equivalence assumption is reasonable. Use causal language carefully, clarifying that IV estimates may provide causal effects only under certain assumptions**	**17**	**Individuals with depression present higher level of inflammation, hence are at increased risk of cardiometabolic disorders irrespective of the age of onset. On the other hand, the phenotypic associations between MD and cardiometabolic traits may partly reflect the genetic overlap between them.**
		**c)**	**Clinical relevance: Discuss whether the results have clinical or public policy relevance, and to what extent they inform effect sizes of possible interventions**	**17**	**Managing cardiometabolic risk in individuals with depression could eventually form part of the clinical practice.**
**17**	**Generalizability**		**Discuss the generalizability of the study results (a) to other populations, (b) across other exposure periods/timings, and (c) across other levels of exposure**	**17**	**Instead of generalising findings about depression risk factors across populations, further studies are needed to understand how genetic and environmental factors contribute to the complex relationships across diverse ancestry groups.**

OTHER INFORMATION

**18**	**Funding**	**Describe sources of funding and the role of funders in the present study and, if applicable, sources of funding for the databases and original study or studies on which the present study is based**	**44-45**	**This study is part of a project that has received funding from Wellcome (212360/ Z/18/Z) and from the European Research Council (ERC) under the European Union's Horizon 2020 research and innovation program (Grant agreement No. 948561).) [...] abbreviated due to length. please see manuscript**
**19**	**Data and data sharing**	**Provide the data used to perform all analyses or report where and how the data can be accessed, and reference these sources in the article. Provide the statistical code needed to reproduce the results in the article, or report whether the code is publicly accessible and if so, where**	**26-27**	**GWAS summary statistics will be made available via the PGC website https://www.med.unc.edu/pgc/download-results/. 23andMe, WHI and JHS do not permit sharing of genome-wide summary statistics. The full GWAS summary statistics for the 23andMe discovery data set will be made available through 23andMe to qualified researchers under an agreement with 23andMe that protects the privacy of the 23andMe participants. Please visit https://research.23andme.com/collaborate/#dataset-access/ for more information and to apply to access the data. Investigators can apply for access to WHI and JHS via dbgap https://www.ncbi.nlm.nih.gov/gap/.<br>We used publicly available software for the analyses. The software used is listed in the Methods section.**
**20**	**Conflicts of Interest**	**All authors should declare all potential conflicts of interest**	**46**	**None**

This checklist is copyrighted by the Equator Network under the Creative Commons Attribution 3.0 Unported (CC BY 3.0) license.

Supplementary Figures

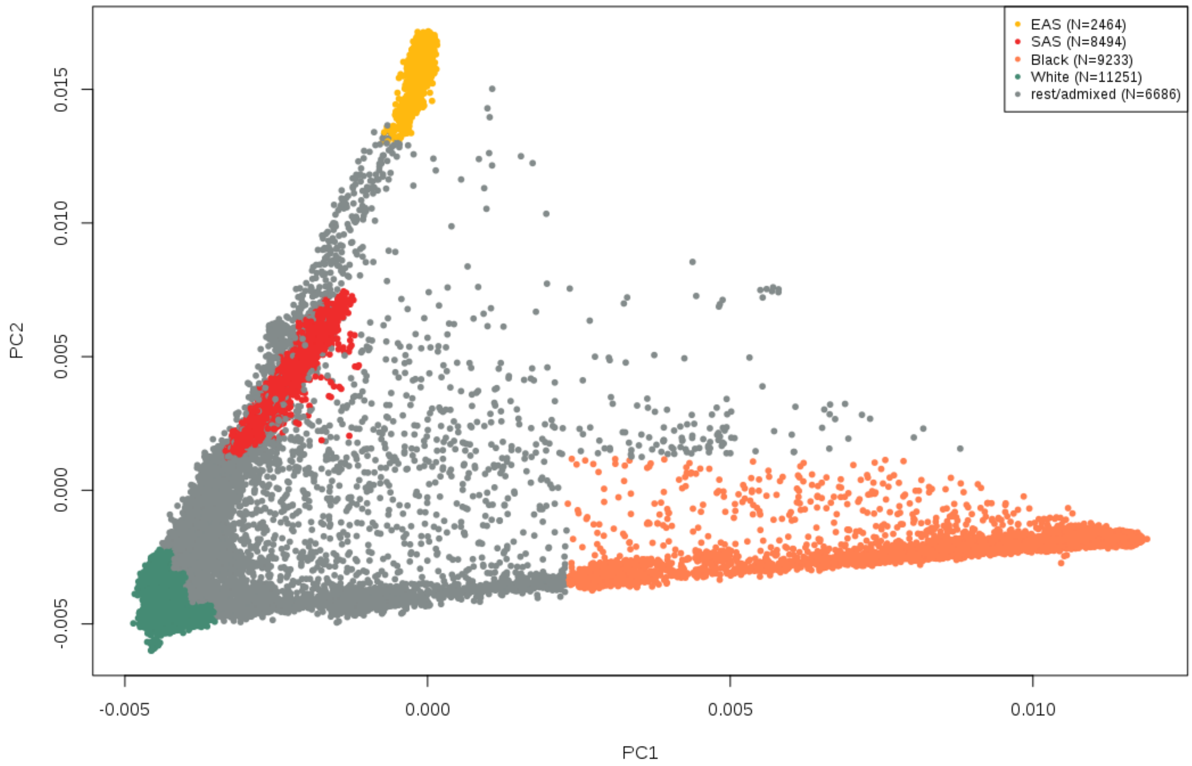

Supplementary Figure 1. Ancestral composition of the UK Biobank study. Each point represents a UK Biobank participant and is placed according to their principal component (PC) scores in the top two principal components. Colours indicate the ancestry group each individual was assigned to.

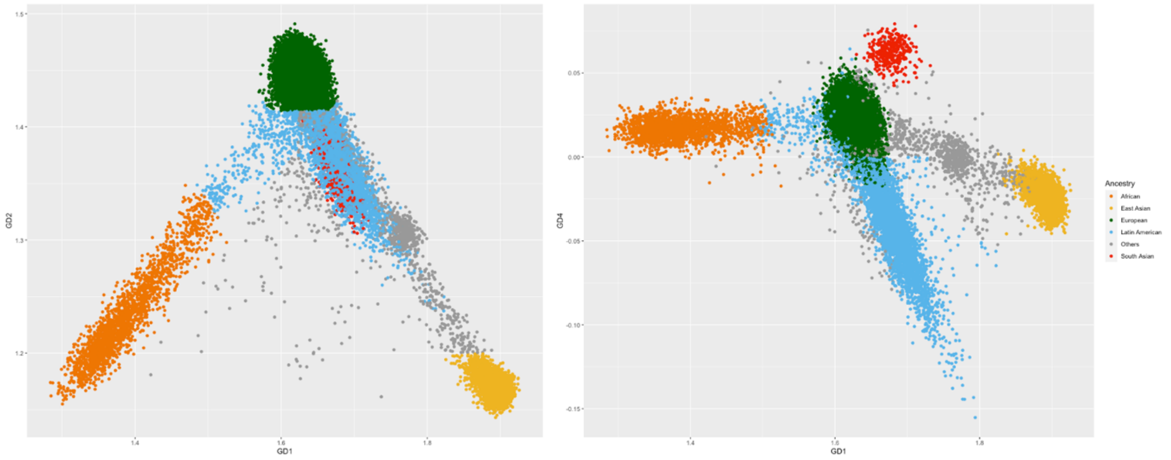

Supplementary Figure 2. Ancestral composition of the GERA study. Each point represents a participant and is placed according to their principal component (PC) scores in the top four principal components. Colours indicate the ancestry/ethnic group each individual was assigned to.

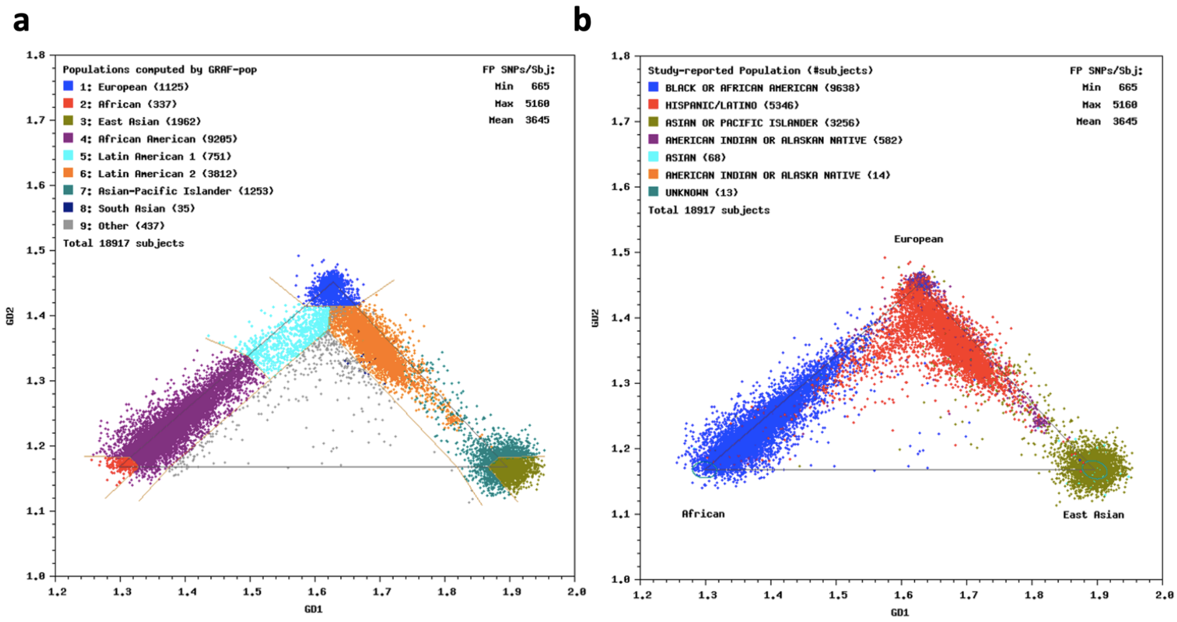

Supplementary Figure 3. Ancestry composition of the WHI Page Study, produced using dbgap's GRAF-pop. Each point represents a participant and is placed according to their principal component (PC) scores in the top four principal components. a) Colours indicate the ancestry/ethnic group each individual was assigned to. b) Colours indicate the study-reported group.

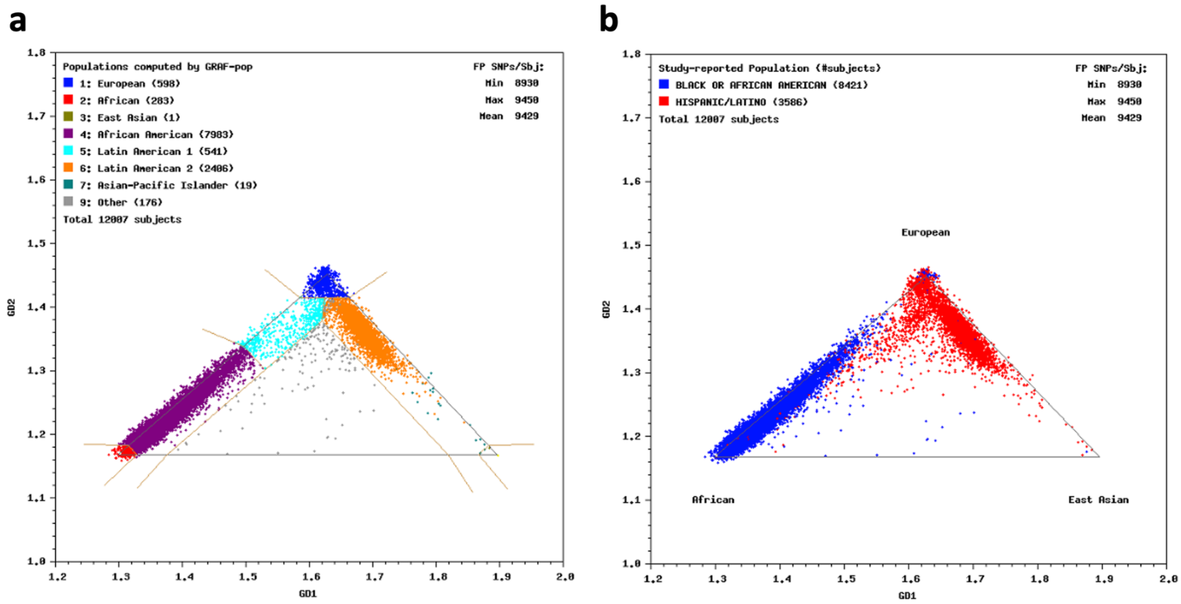

Supplementary Figure 4. Ancestry composition of the WHI Share Study, produced using dbgap's GRAF-pop. Each point represents a participant and is placed according to their principal component (PC) scores in the top four principal components. a) Colours indicate the ancestry/ethnic group each individual was assigned to. b) Colours indicate the study-reported group.

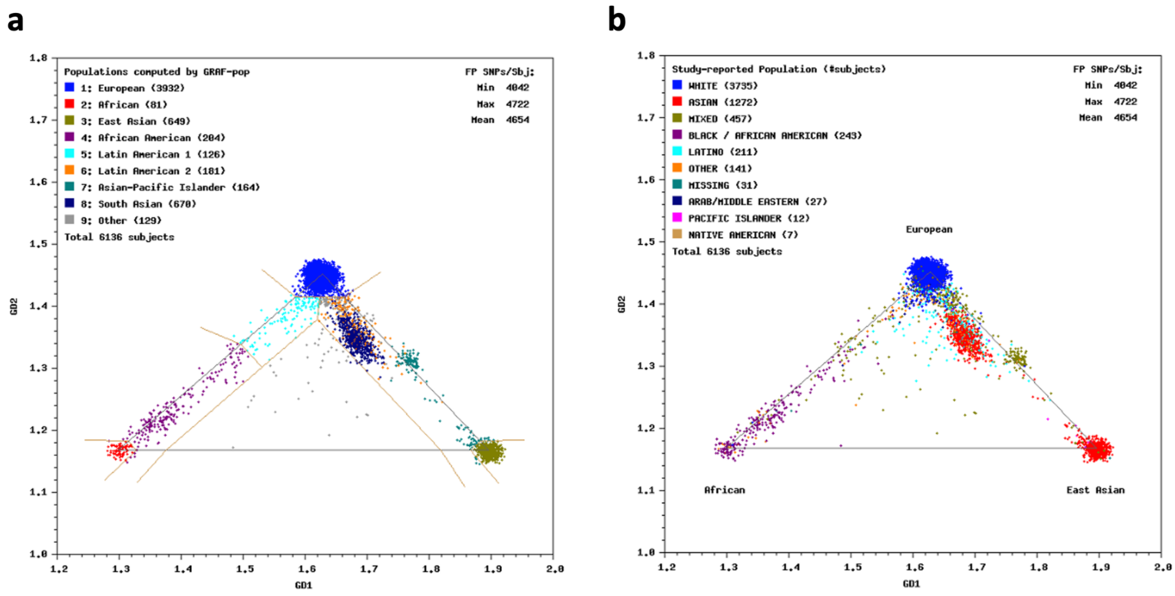

Supplementary Figure 5. Ancestry composition of the IHS study, produced using dbgap's GRAF-pop. Each point represents a participant and is placed according to their principal component (PC) scores in the top four principal components. a) Colours indicate the ancestry/ethnic group each individual was assigned to. b) Colours indicate the study-reported group.

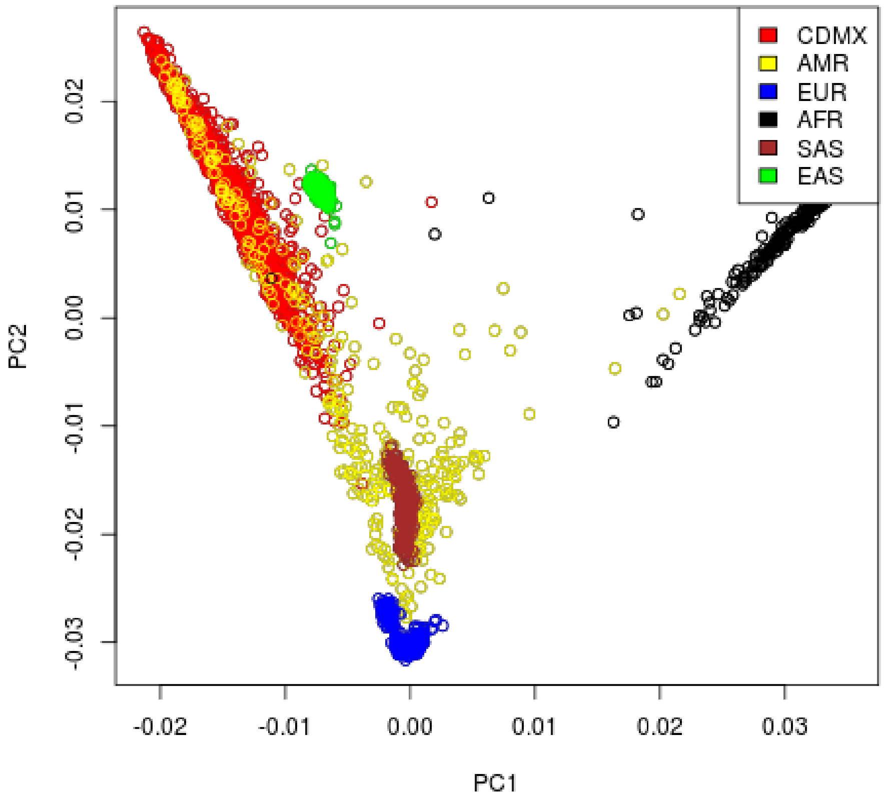

Supplementary Figure 6. Ancestry composition of the MAMHS study. Each point represents a participant and is placed according to their principal component (PC) scores in the top two principal components. Colours indicate the ancestry/ethnic group each individual was assigned to.

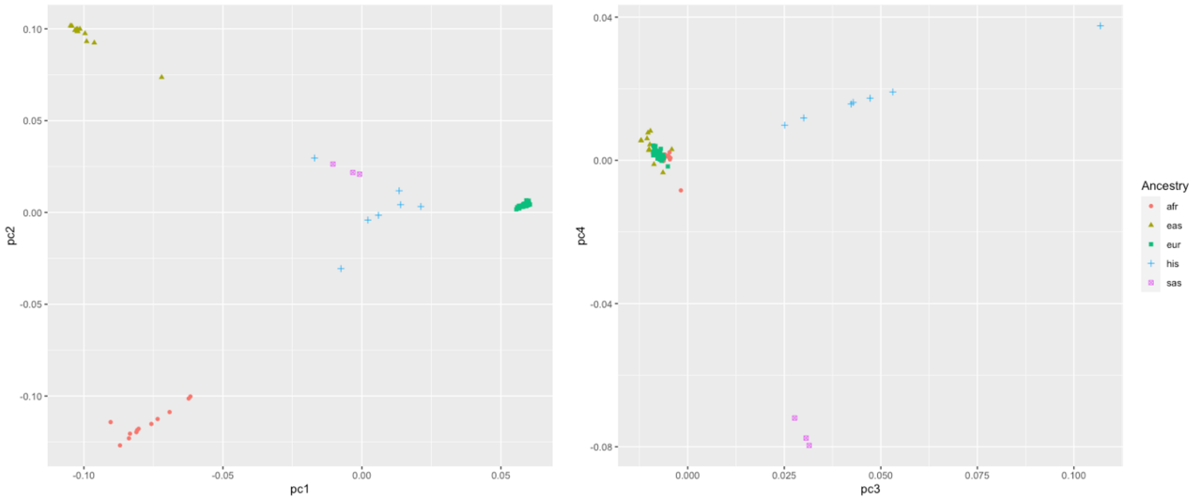

Supplementary Figure 7. Ancestral principal components representing the average ancestry composition for each study included in the meta-analysis, computed using MR-MEGA

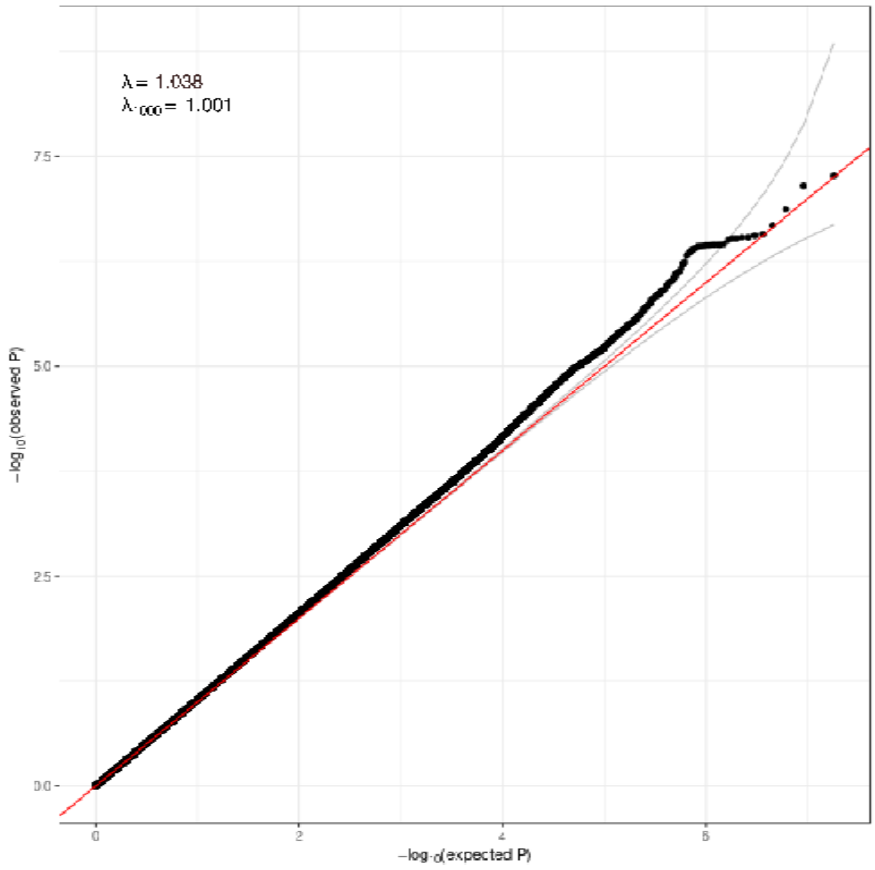

Supplementary Figure 8. QQ plot for genetic associations with major depression in individuals of African ancestry.

λ is an estimate for the inflation and λ_{1000} is the estimate adjusted for sample size.

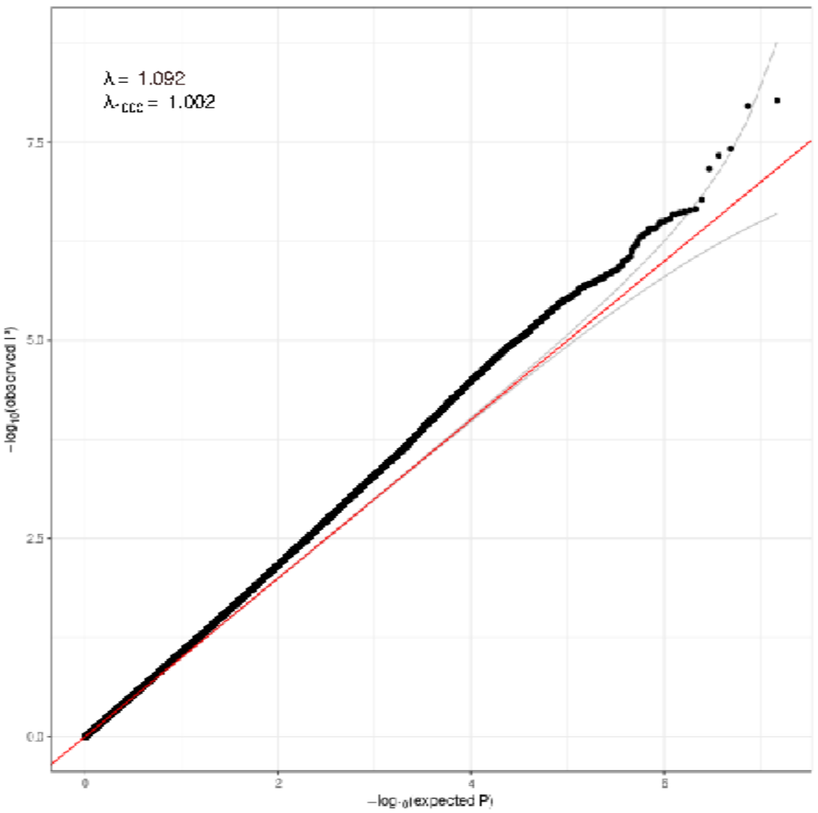

Supplementary Figure 9. QQ plot for genetic associations with major depression in individuals in the Hispanic/Latin American group. **λ** is an estimate for the inflation and $\boldsymbol{\lambda}_{1000}$ is the estimate adjusted for sample size.

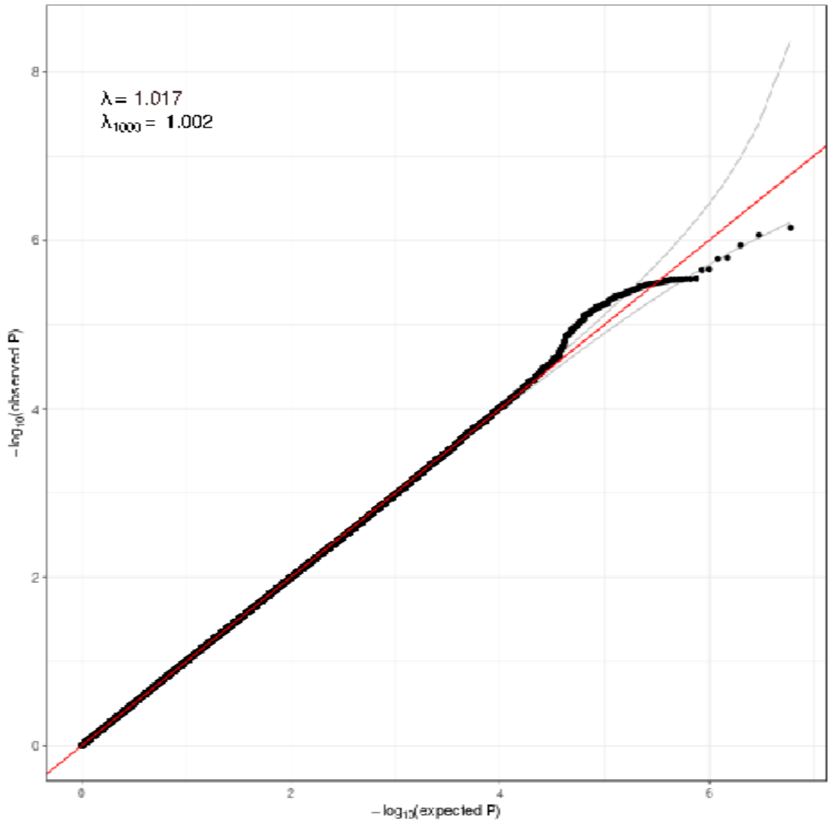

Supplementary Figure 10. QQ plot for genetic associations with major depression in individuals of South Asian ancestry.

λ is an estimate for the inflation and λ_{1000} is the estimate adjusted for sample size.

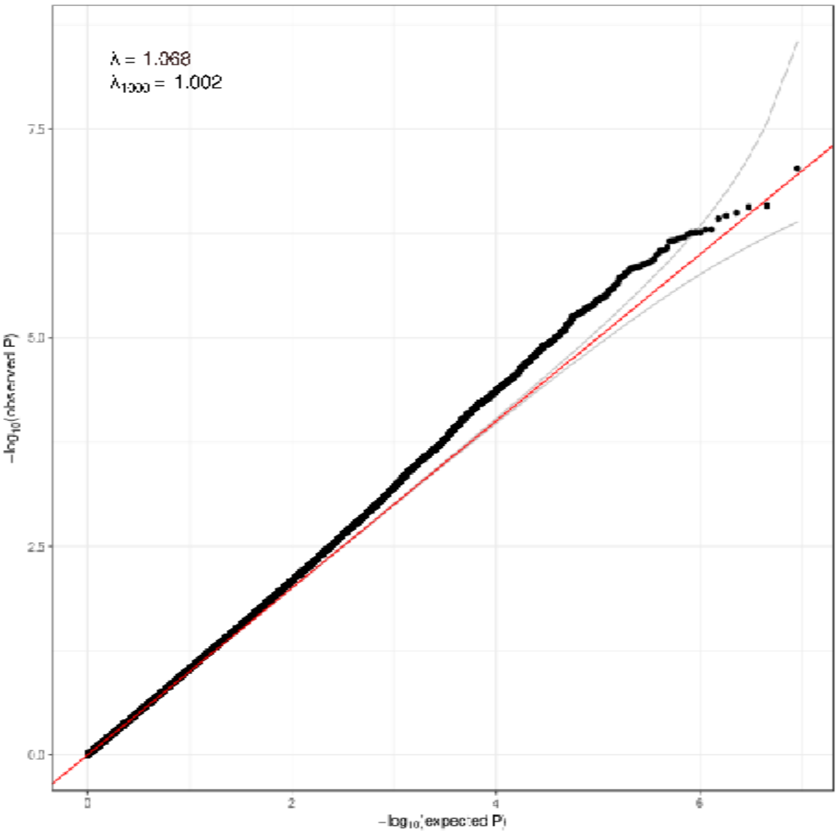

Supplementary Figure 11. QQ plot for genetic associations with major depression in individuals of East Asian ancestry.

λ is an estimate for the inflation and λ_{1000} is the estimate adjusted for sample size.

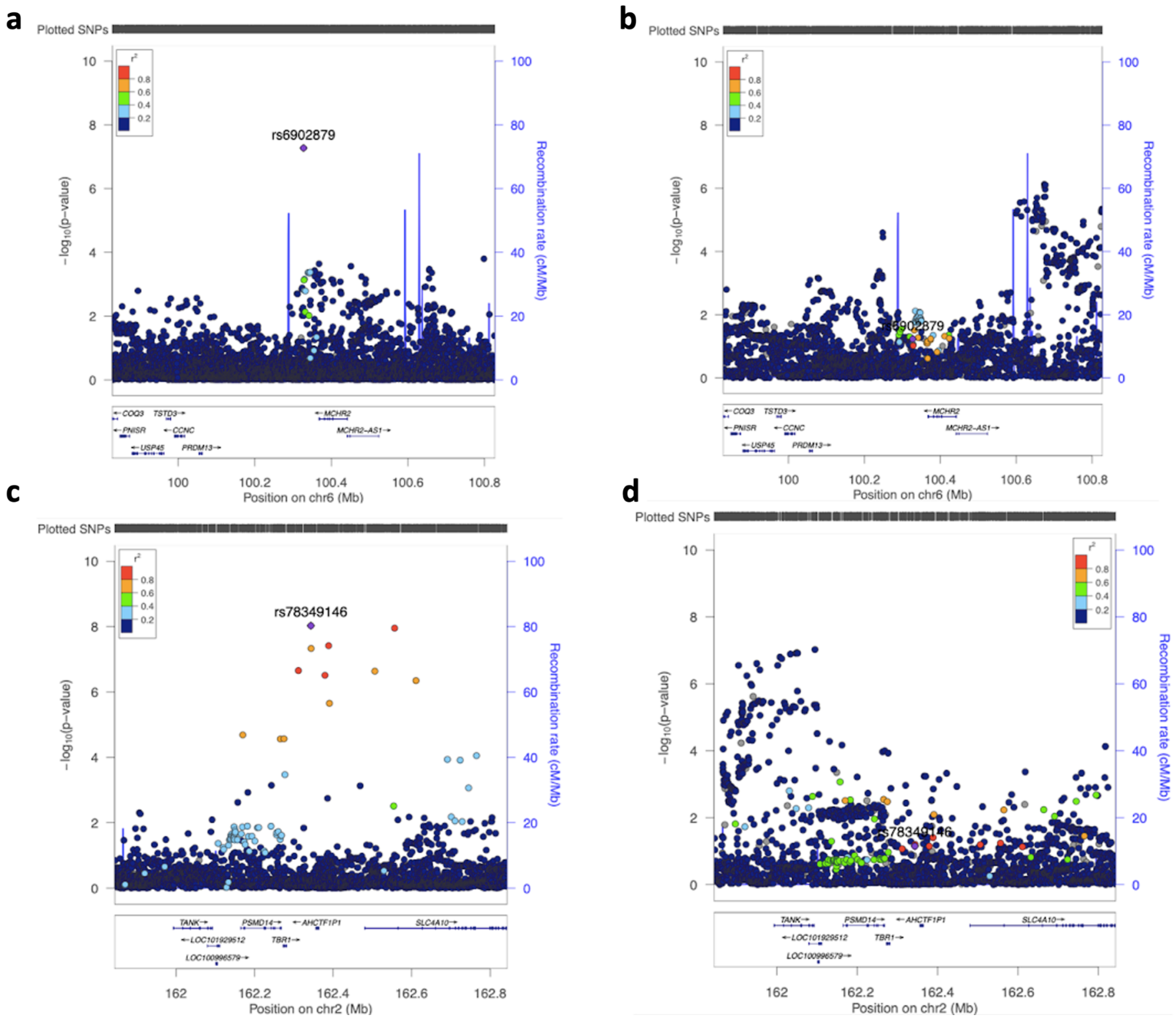

Supplementary Figure 12. Regional association plots for the associations with major depression for two loci from ancestry-specific GWAS. The y-axes shows statistical significance as the $-\log_{10}P$ values of z statistics (two sided-nominal P values) of the association between each SNV and major depression. The x-axes show the chromosomal position (GRCh37). a) Genetic associations for rs6902879 and variants within a 500kb region from the African ancestry meta-analysis; b) Genetic associations for rs6902879 and variants within a 500kb region from the European ancestry meta-analysis; c) Genetic associations of rs78349146 and variants within a 500kb region from the meta-analysis of Hispanic/Latin American individuals; d) Genetic associations for rs78349146 and variants within a 400kb region from the European ancestry meta-analysis.

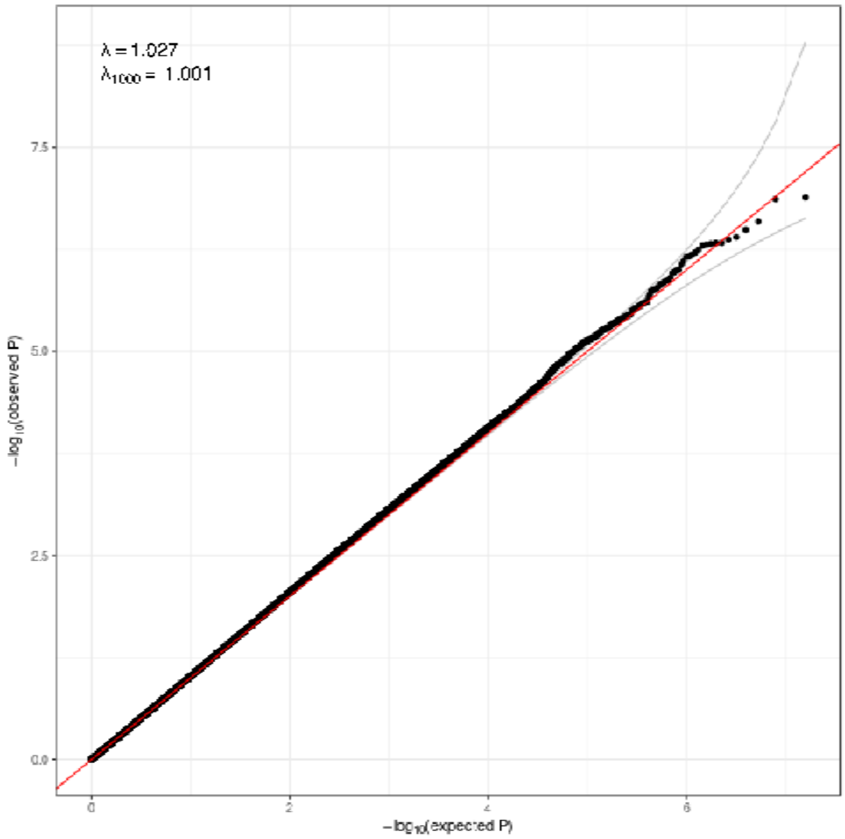

Supplementary Figure 13. QQ plot and for genetic associations with clinical major depression in individuals of African ancestry. **λ** is an estimate for the inflation and **λ_{1000}** is the estimate adjusted for sample size.

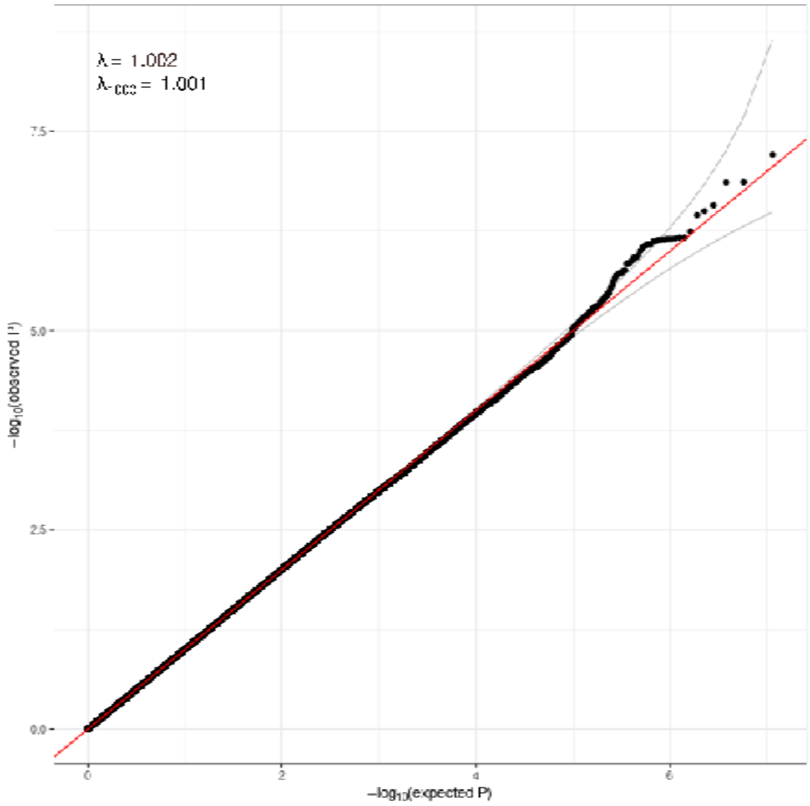

Supplementary Figure 14. QQ plot for genetic associations with clinical major depression in individuals in the Hispanic/Latin American group. **λ** is an estimate for the inflation and **λ_{1000}** is the estimate adjusted for sample size.

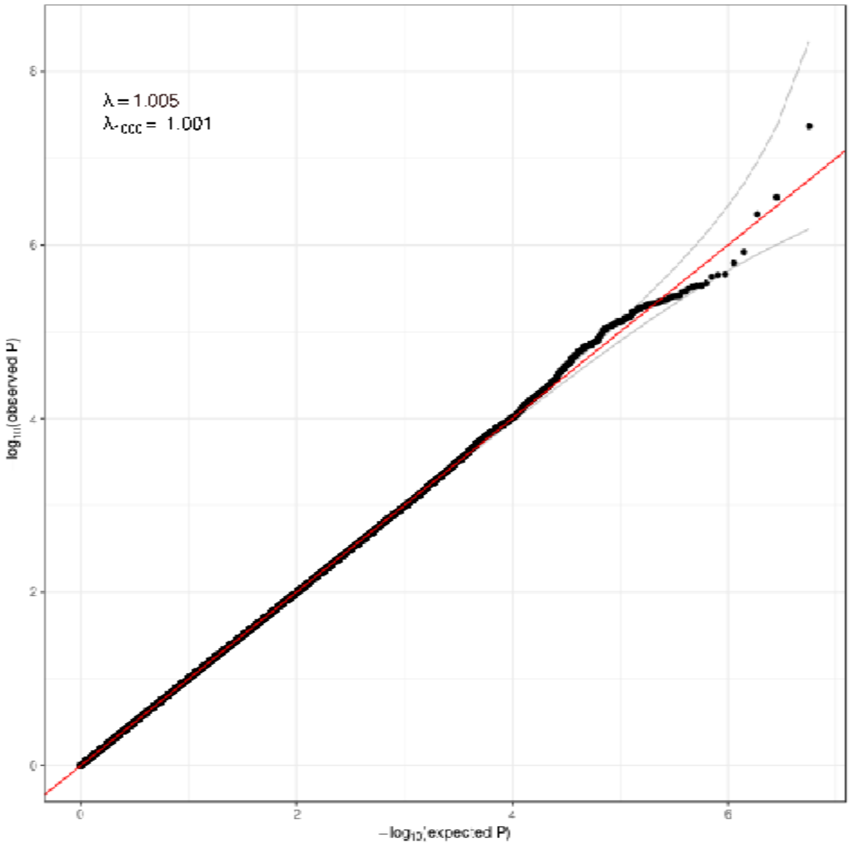

Supplementary Figure 15. QQ plot for genetic associations with clinical major depression in individuals of South Asian ancestry. λ is an estimate for the inflation and λ_{1000} is the estimate adjusted for sample size.

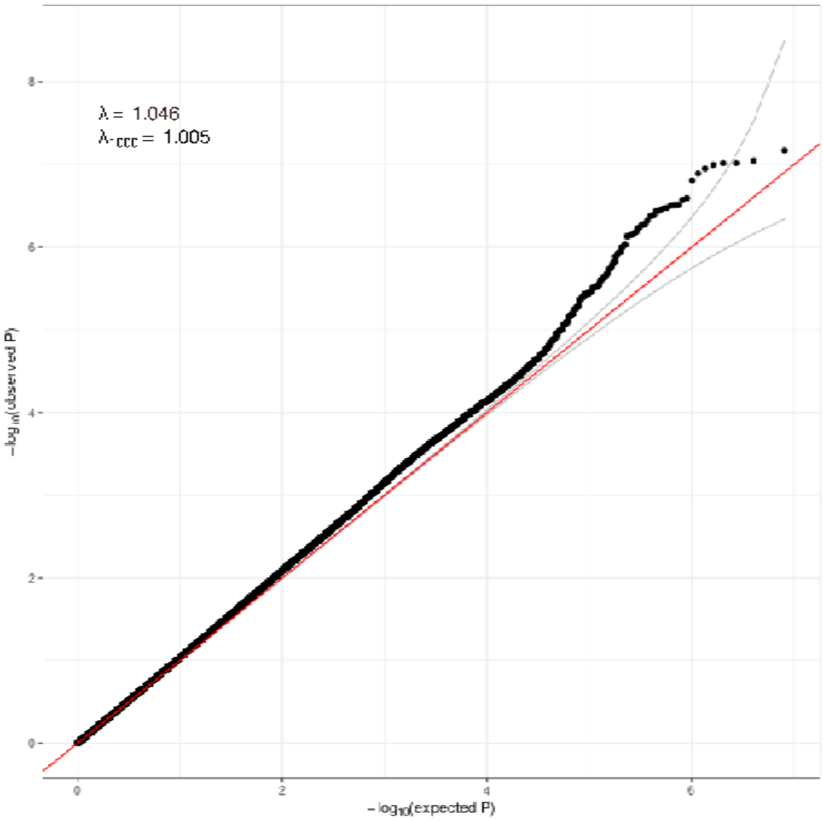

Supplementary Figure 16. QQ plot for genetic associations with clinical major depression in individuals of East Asian ancestry. λ is an estimate for the inflation and λ_{1000} is the estimate adjusted for sample size.

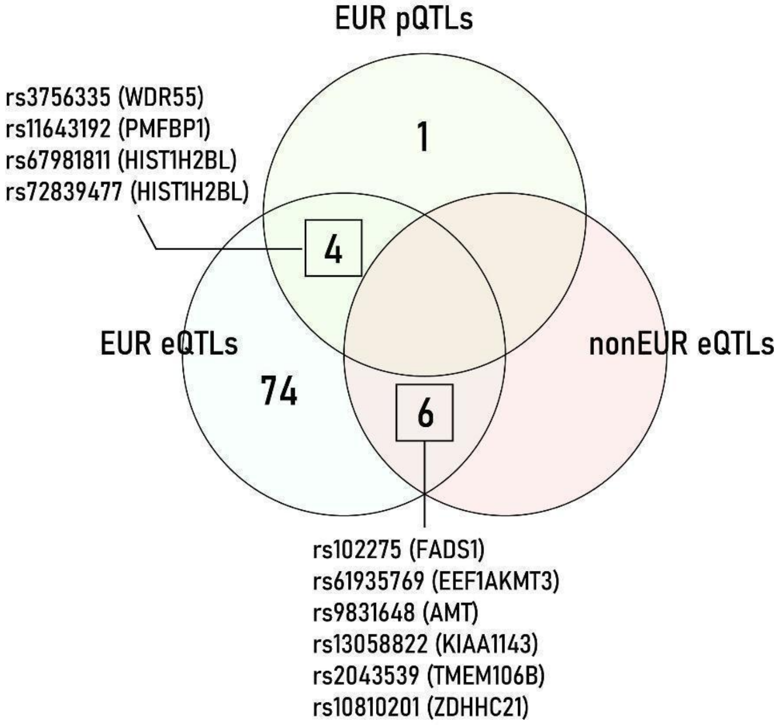

Supplementary Figure 17. Transferability SNPs mapped as QTLs from brain and blood tissue. We show the distribution of the 85 SNPs that are QTLs for brain and blood tissue for European and non-European ancestry studies.

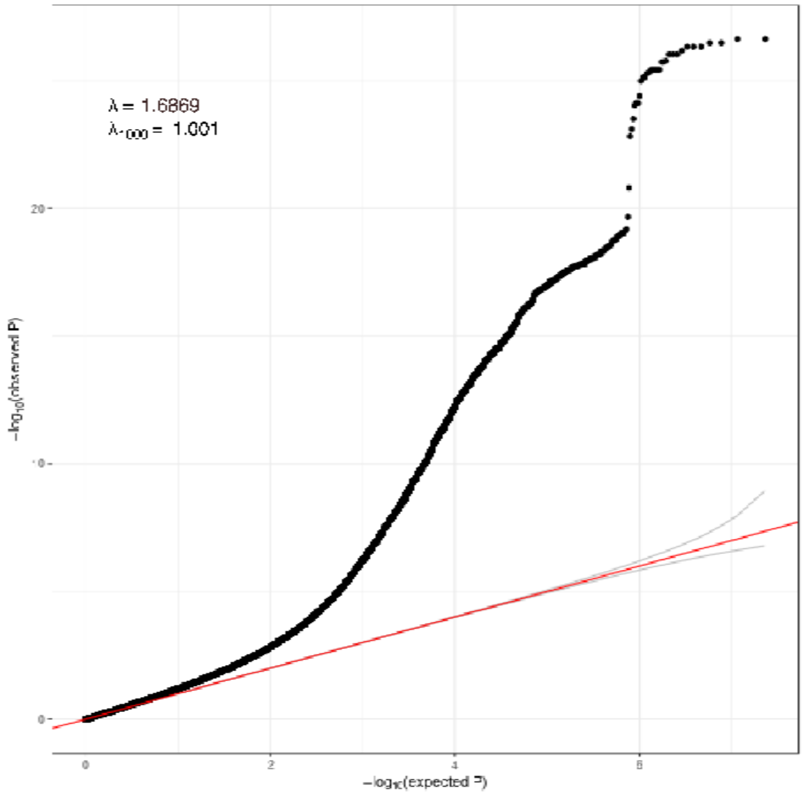

Supplementary Figure 18. QQ plot for genetic associations with major depression in the multi-ancestry meta-analysis. **λ** is an estimate for the inflation and $\boldsymbol{\lambda}_{1000}$ is the estimate adjusted for sample size. Association *P* values have been adjusted by the LDSC intercept of 1.0185.

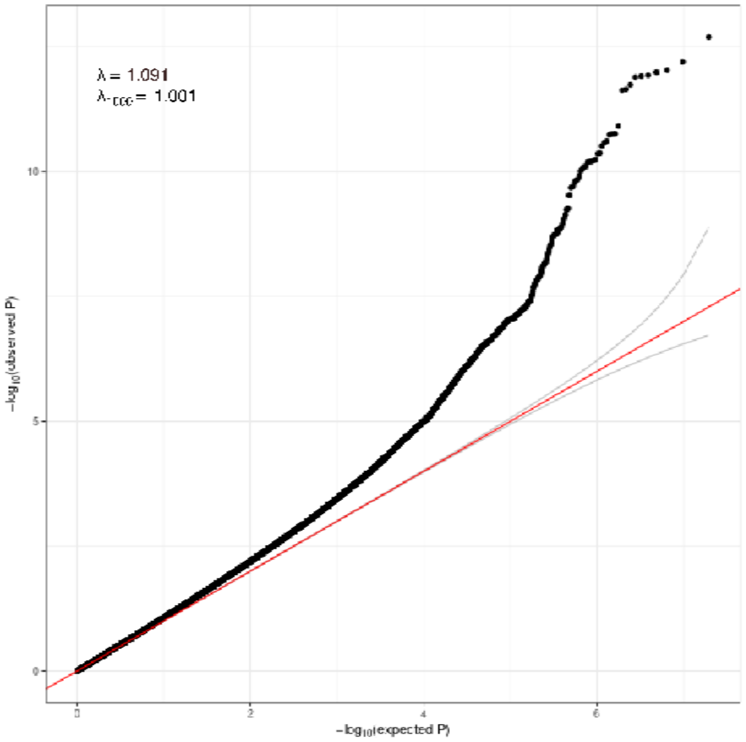

Supplementary Figure 19. QQ plot for genetic associations with clinical major depression in the multi-ancestry meta-analysis. **λ** is an estimate for the inflation and **λ_{1000}** is the estimate adjusted for sample size.

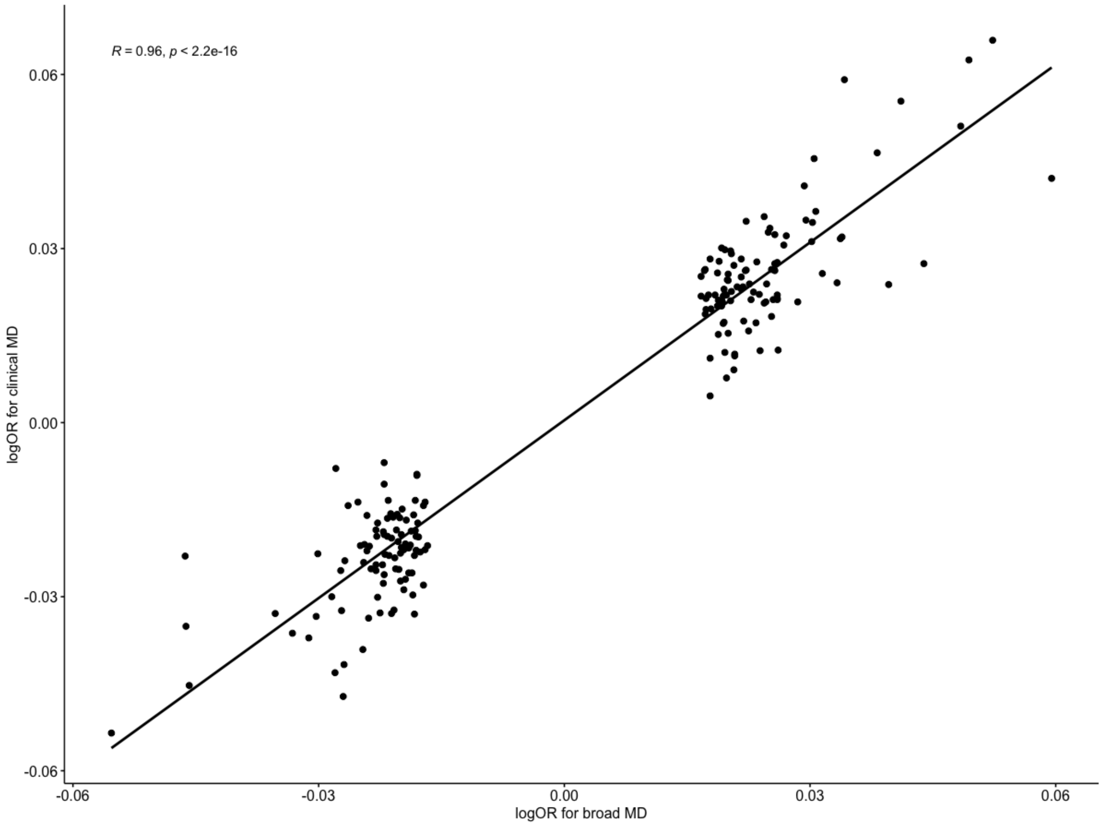

Supplementary Figure 20. Comparison of results for the genome-wide significant variants between broad and clinical depression. Regression coefficients (log odds ratios) for the genome-wide significant loci from the multi-ancestry GWAS for broad major depression on the x-axis and regression coefficients from the analysis based on clinical major depression on the y-axis. A two-sided Pearson correlation analysis was performed to assess the relationship between effect estimates derived from broad depression and clinical depression (R = 0.96, $P = 5.83 \times 10^{-105}$).

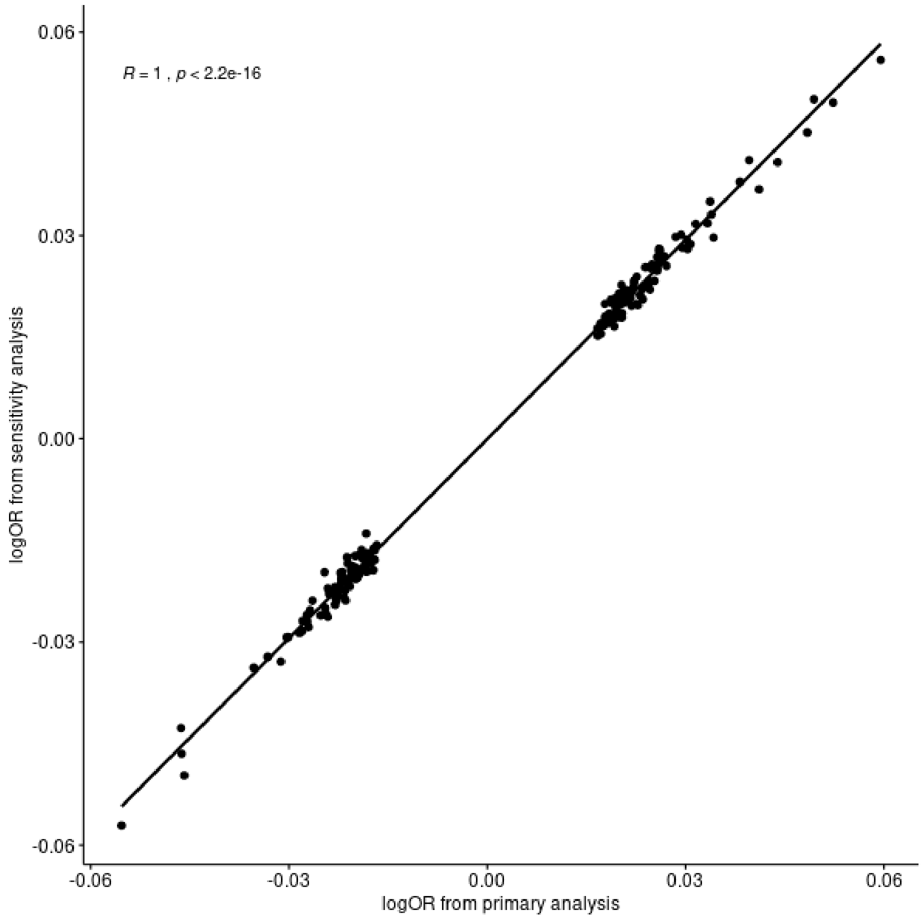

Supplementary Figure 21. Comparison of results for the genome-wide significant variants between the main analysis and a sensitivity analysis excluding studies with extreme case-control ratio. Regression coefficients (log odds ratios) for the genome-wide significant loci from the multi-ancestry GWAS for broad major depression using all studies on the x-axis and regression coefficients from the analysis excluding studies with extreme case-control ratio on the y-axis. A two-sided Pearson correlation analysis was conducted to evaluate the association between effect estimates obtained from the primary meta-analysis and those from the sensitivity meta-analysis (R = 1, P = 2.02 $\times 10^{-231}$).

References

1. Giannakopoulou, O. *et al.* The Genetic Architecture of Depression in Individuals of East Asian Ancestry: A Genome-Wide Association Study. *JAMA Psychiatry* **78**, 1258–1269 (2021).
2. Hyland, A. *et al.* Associations of lifetime active and passive smoking with spontaneous abortion, stillbirth and tubal ectopic pregnancy: a cross-sectional analysis of historical data from the Women's Health Initiative. *Tobacco Control* vol. 24 328–335 Preprint at https://doi.org/10.1136/tobaccocontrol-2013-051458 (2015).
3. Frank, E., Nallamothu, B. K., Zhao, Z. & Sen, S. Political events and mood among young physicians: a prospective cohort study. *BMJ* **367**, l6322 (2019).
4. Ursano, R. J. *et al.* The Army study to assess risk and resilience in servicemembers (Army STARRS). *Psychiatry* **77**, 107–119 (2014).
5. Finer, S. *et al.* Cohort Profile: East London Genes & Health (ELGH), a community-based population genomics and health study in British Bangladeshi and British Pakistani people. *Int. J. Epidemiol.* **49**, 20–21i (2020).
6. Sakaue, S. *et al.* A cross-population atlas of genetic associations for 220 human phenotypes. *Nat. Genet.* **53**, 1415–1424 (2021).
7. Nagai, A. *et al.* Overview of the BioBank Japan Project: Study design and profile. *J. Epidemiol.* **27**, S2–S8 (2017).
8. Akiyama, M. *et al.* Characterizing rare and low-frequency height-associated variants in the Japanese population. *Nat. Commun.* **10**, 4393 (2019).
9. Loh, P.-R., Palamara, P. F. & Price, A. L. Fast and accurate long-range phasing in a UK

Biobank cohort. *Nat. Genet.* **48**, 811–816 (2016).

10. Li, Y., Willer, C. J., Ding, J., Scheet, P. & Abecasis, G. R. MaCH: using sequence and genotype data to estimate haplotypes and unobserved genotypes. *Genet. Epidemiol.* **34**, 816–834 (2010).
11. Gaziano, J. M. *et al.* Million Veteran Program: A mega-biobank to study genetic influences on health and disease. *J. Clin. Epidemiol.* **70**, 214–223 (2016).
12. Hunter-Zinck, H. *et al.* Genotyping Array Design and Data Quality Control in the Million Veteran Program. *Am. J. Hum. Genet.* **106**, 535–548 (2020).
13. Levey, D. F. *et al.* Bi-ancestral depression GWAS in the Million Veteran Program and meta-analysis in >1.2 million individuals highlight new therapeutic directions. *Nat. Neurosci.* **24**, 954–963 (2021).
14. Mitchell, B. L. *et al.* The Australian Genetics of Depression Study: New Risk Loci and Dissecting Heterogeneity Between Subtypes. *Biol. Psychiatry* **92**, 227–235 (2022).
15. Olsen, C. M. *et al.* Cohort profile: the QSkin Sun and Health Study. *Int. J. Epidemiol.* **41**, 929–929i (2012).
16. Zhou, W. *et al.* Efficiently controlling for case-control imbalance and sample relatedness in large-scale genetic association studies. *Nat. Genet.* **50**, 1335–1341 (2018).
17. Banda, Y. *et al.* Characterizing Race/Ethnicity and Genetic Ancestry for 100,000 Subjects in the Genetic Epidemiology Research on Adult Health and Aging (GERA) Cohort. *Genetics* vol. 200 1285–1295 Preprint at https://doi.org/10.1534/genetics.115.178616 (2015).
18. Kvale, M. N. *et al.* Genotyping Informatics and Quality Control for 100,000 Subjects in the Genetic Epidemiology Research on Adult Health and Aging (GERA) Cohort. *Genetics* **200**, 1051–1060 (2015).

19. Hoffmann, T. J. *et al.* Design and coverage of high throughput genotyping arrays optimized for individuals of East Asian, African American, and Latino race/ethnicity using imputation and a novel hybrid SNP selection algorithm. *Genomics* vol. 98 422–430 Preprint at https://doi.org/10.1016/j.ygeno.2011.08.007 (2011).

20. Howie, B. N., Donnelly, P. & Marchini, J. A flexible and accurate genotype imputation method for the next generation of genome-wide association studies. *PLoS Genet.* **5**, e1000529 (2009).

21. Howie, B., Fuchsberger, C., Stephens, M., Marchini, J. & Abecasis, G. R. Fast and accurate genotype imputation in genome-wide association studies through pre-phasing. *Nat. Genet.* **44**, 955–959 (2012).

22. Jin, Y., Schaffer, A. A., Feolo, M., Holmes, J. B. & Kattman, B. L. GRAF-pop: A Fast Distance-Based Method To Infer Subject Ancestry from Multiple Genotype Datasets Without Principal Components Analysis. *G3* **9**, 2447–2461 (2019).

23. Davis, S. K. *et al.* Association of ADIPOQ gene with type 2 diabetes and related phenotypes in African American men and women: the Jackson Heart Study. *BMC Genet.* **16**, 147 (2015).

24. Wyatt, S. B. *et al.* A community-driven model of research participation: the Jackson Heart Study Participant Recruitment and Retention Study. *Ethn. Dis.* **13**, 438–455 (2003).

25. Manichaikul, A. *et al.* Robust relationship inference in genome-wide association studies. *Bioinformatics* **26**, 2867–2873 (2010).

26. Donald, K. A. *et al.* Drakenstein Child Health Study (DCHS): investigating determinants of early child development and cognition. *BMJ Paediatr Open* **2**, e000282 (2018).

27. Stein, D. J. *et al.* Investigating the psychosocial determinants of child health in Africa:

The Drakenstein Child Health Study. *J. Neurosci. Methods* **252**, 27–35 (2015).

28. Zar, H. J., Barnett, W., Myer, L., Stein, D. J. & Nicol, M. P. Investigating the early-life determinants of illness in Africa: the Drakenstein Child Health Study. *Thorax* vol. 70 592–594 Preprint at https://doi.org/10.1136/thoraxjnl-2014-206242 (2015).
29. Koen, N. *et al.* Intimate partner violence: associations with low infant birthweight in a South African birth cohort. *Metabolic Brain Disease* vol. 29 281–299 Preprint at https://doi.org/10.1007/s11011-014-9525-4 (2014).
30. Roden, D. M. *et al.* Development of a large-scale de-identified DNA biobank to enable personalized medicine. *Clin. Pharmacol. Ther.* **84**, 362–369 (2008).
31. Ruderfer, D. M. *et al.* Significant shared heritability underlies suicide attempt and clinically predicted probability of attempting suicide. *Mol. Psychiatry* **25**, 2422–2430 (2020).
32. Dennis, J. *et al.* Genetic risk for major depressive disorder and loneliness in sex-specific associations with coronary artery disease. *Mol. Psychiatry* **26**, 4254–4264 (2021).
33. Lavange, L. M. *et al.* Sample design and cohort selection in the Hispanic Community Health Study/Study of Latinos. *Ann. Epidemiol.* **20**, 642–649 (2010).
34. Sorlie, P. D. *et al.* Design and implementation of the Hispanic Community Health Study/Study of Latinos. *Ann. Epidemiol.* **20**, 629–641 (2010).
35. Conomos, M. P. *et al.* Genetic Diversity and Association Studies in US Hispanic/Latino Populations: Applications in the Hispanic Community Health Study/Study of Latinos. *Am. J. Hum. Genet.* **98**, 165–184 (2016).
36. Dunn, E. C. *et al.* Genome-wide association study of depressive symptoms in the Hispanic Community Health Study/Study of Latinos. *Journal of Psychiatric Research* vol. 99 167–176 Preprint at https://doi.org/10.1016/j.jpsychires.2017.12.010 (2018).

37. Gogarten, S. M. *et al.* Genetic association testing using the GENESIS R/Bioconductor package. *Bioinformatics* vol. 35 5346–5348 Preprint at https://doi.org/10.1093/bioinformatics/btz567 (2019).

38. Uddin, M. *et al.* Epigenetic and immune function profiles associated with posttraumatic stress disorder. *Proceedings of the National Academy of Sciences* vol. 107 9470–9475 Preprint at https://doi.org/10.1073/pnas.0910794107 (2010).

39. Kroenke, K., Spitzer, R. L. & Williams, J. B. The PHQ-9: validity of a brief depression severity measure. *J. Gen. Intern. Med.* **16**, 606–613 (2001).

40. American Psychiatric Association, American Psychiatric Association Staff, Asociación Amerciana de Psiquiatría, Davenport, J. H. & American Psychiatric Association. Task Force on DSM-IV. *Diagnostic and Statistical Manual of Mental Disorders: DSM-IV*. (American Psychiatric Publishing, 1994).

41. Nievergelt, C. M. *et al.* International meta-analysis of PTSD genome-wide association studies identifies sex- and ancestry-specific genetic risk loci. *Nat. Commun.* **10**, 4558 (2019).

42. Storr, C. L., Or, F., Eaton, W. W. & Ialongo, N. Genetic research participation in a young adult community sample. *Journal of Community Genetics* vol. 5 363–375 Preprint at https://doi.org/10.1007/s12687-014-0191-3 (2014).

43. Ialongo, N. S. *et al.* Proximal Impact of Two First-Grade Preventive Interventions on the Early Risk Behaviors for Later Substance Abuse, Depression, and Antisocial Behavior. *American Journal of Community Psychology* vol. 27 599–641 Preprint at https://doi.org/10.1023/a:1022137920532 (1999).

44. Uhl, G. R. *et al.* Smoking quit success genotype score predicts quit success and distinct patterns of developmental involvement with common addictive substances. *Mol.*

Psychiatry **19**, 50–54 (2014).

45. Das, S. *et al.* Next-generation genotype imputation service and methods. *Nat. Genet.* **48**, 1284–1287 (2016).
46. Benjet, C. *et al.* Descriptive epidemiology of chronic childhood adversity in Mexican adolescents. *J. Adolesc. Health* **45**, 483–489 (2009).
47. Martinez-Levy, G. A. *et al.* Suicidal ideation and planning among Mexican adolescents are associated with depression polygenic risk scores. *Am. J. Med. Genet. B Neuropsychiatr. Genet.* **186**, 476–484 (2021).
48. Shen, H. *et al.* Polygenic prediction and GWAS of depression, PTSD, and suicidal ideation/self-harm in a Peruvian cohort. *Neuropsychopharmacology* vol. 45 1595–1602 Preprint at https://doi.org/10.1038/s41386-020-0603-5 (2020).
49. Barrios, Y. V. *et al.* Childhood abuse and early menarche among Peruvian women. *J. Adolesc. Health* **56**, 197–202 (2015).
50. 1000 Genomes Project Consortium *et al.* A global reference for human genetic variation. *Nature* **526**, 68–74 (2015).
51. Conomos, M. P., Reiner, A. P., Weir, B. S. & Thornton, T. A. Model-free Estimation of Recent Genetic Relatedness. *Am. J. Hum. Genet.* **98**, 127–148 (2016).
52. Skrivankova, V. W. *et al.* Strengthening the reporting of observational studies in epidemiology using mendelian randomisation (STROBE-MR): explanation and elaboration. *BMJ* **375**, n2233 (2021).
53. Skrivankova, V. W. *et al.* Strengthening the Reporting of Observational Studies in Epidemiology Using Mendelian Randomization: The STROBE-MR Statement. *JAMA* **326**, 1614–1621 (2021).
54. Howard, D. M. *et al.* Genome-wide meta-analysis of depression identifies 102

independent variants and highlights the importance of the prefrontal brain regions. *Nat. Neurosci.* **22**, 343–352 (2019).

55. Willer, C. J., Li, Y. & Abecasis, G. R. METAL: fast and efficient meta-analysis of genomewide association scans. *Bioinformatics* **26**, 2190–2191 (2010).
56. Turcot, V. *et al.* Protein-altering variants associated with body mass index implicate pathways that control energy intake and expenditure in obesity. *Nat. Genet.* **50**, 26–41 (2018).
57. Chen, J. *et al.* The trans-ancestral genomic architecture of glycemic traits. *Nat. Genet.* **53**, 840–860 (2021).
58. Graham, S. E. *et al.* The power of genetic diversity in genome-wide association studies of lipids. *Nature* **600**, 675–679 (2021).
59. Hemani, G. *et al.* The MR-Base platform supports systematic causal inference across the human phenome. *Elife* **7**, (2018).
60. Wray, N. R. *et al.* Genome-wide association analyses identify 44 risk variants and refine the genetic architecture of major depression. *Nat. Genet.* **50**, 668–681 (2018).
