# Supplementary material for: Multi-ancestry genome-wide association study of major depression aids locus discovery, fine mapping, gene prioritization and causal inference
Source: Nat Genet. 2024 Jan 4;56(2):222–33. doi: 10.1038/s41588-023-01596-4 (PMC10864182; doi:10.1038/s41588-023-01596-4)
Supplement: Supplementary file 2 — Reporting Summary [file 41588_2023_1596_MOESM2_ESM.pdf]

nature portfolio

Corresponding author(s): Karoline Kuchenbaecker

Last updated by author(s): Sep 27, 2023

Reporting Summary

Nature Portfolio wishes to improve the reproducibility of the work that we publish. This form provides structure for consistency and transparency in reporting. For further information on Nature Portfolio policies, see our Editorial Policies and the Editorial Policy Checklist.

Statistics

For all statistical analyses, confirm that the following items are present in the figure legend, table legend, main text, or Methods section.

n/a	Confirmed	
☐	☒	The exact sample size (n) for each experimental group/condition, given as a discrete number and unit of measurement
☐	☒	A statement on whether measurements were taken from distinct samples or whether the same sample was measured repeatedly
☐	☒	The statistical test(s) used AND whether they are one- or two-sided<br>*Only common tests should be described solely by name; describe more complex techniques in the Methods section.*
☐	☒	A description of all covariates tested
☐	☒	A description of any assumptions or corrections, such as tests of normality and adjustment for multiple comparisons
☐	☒	A full description of the statistical parameters including central tendency (e.g. means) or other basic estimates (e.g. regression coefficient) AND variation (e.g. standard deviation) or associated estimates of uncertainty (e.g. confidence intervals)
☐	☒	For null hypothesis testing, the test statistic (e.g. F, t, r) with confidence intervals, effect sizes, degrees of freedom and P value noted<br>*Give P values as exact values whenever suitable.*
☒	☐	For Bayesian analysis, information on the choice of priors and Markov chain Monte Carlo settings
☒	☐	For hierarchical and complex designs, identification of the appropriate level for tests and full reporting of outcomes
☐	☒	Estimates of effect sizes (e.g. Cohen's d, Pearson's r), indicating how they were calculated

Our web collection on statistics for biologists contains articles on many of the points above.

Software and code

Policy information about availability of computer code

Data collection	Data collection not part of this study. No software was used for data collection.
Data analysis	Mixed-effect models were used in the association analysis for CKB, BioME, Genes & Health with SAIGE (version 0.36.1, version 0.37, or version 0.39). The CONVERGE study initially conducted mixed-effect model GWA tests with FastLMM (version 2.06.20130802), followed by PLINK logistic regressions to retrieve logORs. For the CONVERGE study, the logORs and standard errors from PLINK were used in our meta-analysis. The HCHS/SOL implemented mixed-effect model GWA tests to adjust for population structure and relatedness with depression as binary outcome and was run using GENESIS.<br>We implemented inverse-variance weighted fixed-effect meta-analyses using METAL (version 2011-03-25) and a multi ancestry meta-regression using MR-MEGA (v.0.2).<br>To identify independent association signals, the GCTA (version 1.92.0 beta2) forward selection and backward elimination process (command 'cojo-slt') were applied using the summary statistics from the multi-ancestry meta-analysis, with a multi ancestry LD reference panel.<br>We performed colocalization between genetic associations with MD and gene expression in brain and blood tissues from samples of European and African ancestry and Hispanic/Latinx participants using coloc R package (version).<br>The summary statistic from the multi-ancestry meta-analysis was first annotated with FUMA (v1.3.7).<br>Gene-based association analyses were implemented using Multi-marker Analysis of GenoMic Annotation (MAGMA, v1.08) and Hi-C coupled MAGMA (H-MAGMA).<br>To perform a transcriptome-wide association study (TWAS), the FUSION software was used.<br>We performed a bi-directional two-sample MR analysis using the TwoSampleMR R package (v0.5.6, https://mrcieu.github.io/TwoSampleMR/index.html).<br>We estimated trans-ancestry genetic correlations using POPCORN v1.029,55,64. Pairwise correlations were calculated between each

combination of the 5 major ancestry/ethnic groups (i.e. African, European, East Asian, Hispanic/Latinx and South Asian) for broad depression and clinical depression separately.

For manuscripts utilizing custom algorithms or software that are central to the research but not yet described in published literature, software must be made available to editors and reviewers. We strongly encourage code deposition in a community repository (e.g. GitHub). See the Nature Portfolio guidelines for submitting code & software for further information.

Data

Policy information about availability of data

All manuscripts must include a data availability statement. This statement should provide the following information, where applicable:

- Accession codes, unique identifiers, or web links for publicly available datasets
- A description of any restrictions on data availability
- For clinical datasets or third party data, please ensure that the statement adheres to our policy

GWAS summary statistics will be made available via the PGC website https://www.med.unc.edu/pgc/download-results/. Dataset identifier: 'mdd2023diverse'. 23andMe, WHI and JHS do not permit sharing of genome-wide summary statistics. The full GWAS summary statistics for the 23andMe discovery data set will be made available through 23andMe to qualified researchers under an agreement with 23andMe that protects the privacy of the 23andMe participants. Please visit https://research.23andme.com/collaborate/#dataset-access/ for more information and to apply to access the data. Investigators can apply for access to WHI and JHS via dbgap https://www.ncbi.nlm.nih.gov/gap/.

Human research participants

Policy information about studies involving human research participants and Sex and Gender in Research.

Reporting on sex and gender

We used biological sex in the study. It was determined based on the participants' genotypes.

Population characteristics

The population characteristics of participants across multiple studies are as follows:

CKB Study: Mean age is 52.2 years (SD=10.7), with a 59.5% female cohort.
CONVERGE Study: Mean age is 46.1 years, with an entirely female cohort.
Taiwan Study: Mean age is 49.2 years (SD=11.3), and 57.5% are female.
WHI Study: For different ancestries, the mean ages are as follows: 62.7 years (SD=7.5) for East Asians, 61.5 years (SD=7.1) for Africans, and 60.3 years (SD=6.7) for Hispanic/Latin Americans. The cohort is 100% female.
IHS Study: Mean ages by ancestry are 27.4 years (SD=2.4) for East Asians, 27.8 years (SD=2.7) for Africans, and 26.6 years (SD=2.1) for South Asians, with female proportions of 54.8%, 63.3%, and 46.9%, respectively.
UKB Study: Mean ages by ancestry are 52.1 years (SD=7.3) for East Asians, 50.7 years (SD=7.4) for Africans, and 53.0 years (SD=8.3) for South Asians. The cohort has 72.1%, 61%, and 43.8% females, respectively.
Army-STARRS Study: Mean ages by ancestry are 24.5 years (SD=6.3) for East Asians, 23.5 years (SD=5.7) for Africans, and 22.8 years (SD=5.1) for Hispanic/Latin Americans, with female proportions of 12.5%, 21.4%, and 15.4%, respectively.
BioMe Study: Mean age is 58.9 years, with 58.7% females.
BBJ Study: Mean age is 63.0 years, with 46.3% females.
AGDS Study: Mean age is 44.1 years (SD=15.1), with 75.1% females.
IHS Study: Mean age is 55.2 years (SD=12.2), with 63.6% females.
DCHS Study: Mean age is 26.4 years (SD=5.6), with an entirely female cohort.
HCHS/SOL Study: Mean age is 46 years (SD=14), with 59% females.
DNHS Study: Mean age is 53.2 years (SD=16.6), with 58.3% females.
PIRC Study: Mean age is 29 years, with 58.5% females.
MAMHS Study: Mean age is 14.28 years, with 68.8% females.
ProMIS Study: Mean age is 28.2 years (SD=6.3), with an entirely female cohort.

Further details on population characteristics are provided in Supplementary Table 1, titled "Cohort Summary."

Recruitment

We provide detailed descriptions of the 21 cohorts included in this study in the supplementary material.

Ethics oversight

Each of the cohorts included was approved by a relevant ethics review board and we have listed the details in the manuscript.

Note that full information on the approval of the study protocol must also be provided in the manuscript.

Field-specific reporting

Please select the one below that is the best fit for your research. If you are not sure, read the appropriate sections before making your selection.

☒ Life sciences ☐ Behavioural & social sciences ☐ Ecological, evolutionary & environmental sciences

For a reference copy of the document with all sections, see nature.com/documents/nr-reporting-summary-flat.pdf

Life sciences study design

All studies must disclose on these points even when the disclosure is negative.

Sample size	To determine sample size we added up the number of participants of each study that was included in a given analysis.
Data exclusions	We restricted the downstream analysis to genetic variants with imputation accuracy info score of 0.7 or higher and effective allele count (2*maf*(1-maf)*N*R2) of 50 or higher. For study of small sample size, we instead required a minor allele frequency of no less than 0.05. The alleles for indels were re-coded as "I" for the longer allele and "D" for the shorter one. Indels of different patterns at the same position were removed.
Replication	All available cohorts of major depression cases and controls were included in the primary multi-ancestry meta-analysis and therefore we do not perform replication for significant loci we identified from the multi-ancestry meta-analysis in independent cohorts.<br>We tested replication of previously identified loci linked to depression from European ancestry across non-European ancestry groups and this is described in the manuscript as transferability. Add some more information as requested by editors.
Randomization	This was a genetic association study. Allocation by genotype.
Blinding	This was a genetic association study, ie observational design. So no blinding was used.

Reporting for specific materials, systems and methods

We require information from authors about some types of materials, experimental systems and methods used in many studies. Here, indicate whether each material, system or method listed is relevant to your study. If you are not sure if a list item applies to your research, read the appropriate section before selecting a response.

Materials & experimental systems

n/a	Involved in the study	
☒	☐	Antibodies
☒	☐	Eukaryotic cell lines
☒	☐	Palaeontology and archaeology
☒	☐	Animals and other organisms
☒	☐	Clinical data
☒	☐	Dual use research of concern

Methods

n/a	Involved in the study	
☒	☐	ChIP-seq
☒	☐	Flow cytometry
☒	☐	MRI-based neuroimaging

March 2021
